# High efficient multisites genome editing in allotetraploid cotton (*Gossypium hirsutum*) using CRISPR/Cas9 system

**DOI:** 10.1111/pbi.12755

**Published:** 2017-06-20

**Authors:** Pengcheng Wang, Jun Zhang, Lin Sun, Yizan Ma, Jiao Xu, Sijia Liang, Jinwu Deng, Jiafu Tan, Qinghua Zhang, Lili Tu, Henry Daniell, Shuangxia Jin, Xianlong Zhang

**Affiliations:** ^1^ National Key Laboratory of Crop Genetic Improvement Huazhong Agricultural University Wuhan Hubei China; ^2^ Department of Biochemistry School of Dental Medicine University of Pennsylvania Philadelphia PA USA

**Keywords:** cotton, allotetraploid, genome editing, CRISPR/Cas9, high‐throughput sequencing

## Abstract

*Gossypium hirsutum* is an allotetraploid with a complex genome. Most genes have multiple copies that belong to At and Dt subgenomes. Sequence similarity is also very high between gene homologues. To efficiently achieve site/gene‐specific mutation is quite needed. Due to its high efficiency and robustness, the CRISPR (clustered regularly interspaced short palindromic repeats)/Cas9 system has exerted broad site‐specific genome editing from prokaryotes to eukaryotes. In this study, we utilized a CRISPR/Cas9 system to generate two sgRNAs in a single vector to conduct multiple sites genome editing in allotetraploid cotton. An exogenously transformed gene *Discosoma red fluorescent protein2*(*DsRed2*) and an endogenous gene *GhCLA1* were chosen as targets. The *DsRed2‐*edited plants in T0 generation reverted its traits to wild type, with vanished red fluorescence the whole plants. Besides, the mutated phenotype and genotype were inherited to their T1 progenies. For the endogenous gene *GhCLA1*, 75% of regenerated plants exhibited albino phenotype with obvious nucleotides and DNA fragments deletion. The efficiency of gene editing at each target site is 66.7–100%. The mutation genotype was checked for both genes with Sanger sequencing. Barcode‐based high‐throughput sequencing, which could be highly efficient for genotyping to a population of mutants, was conducted in *GhCLA1*‐edited T0 plants and it matched well with Sanger sequencing results. No off‐target editing was detected at the potential off‐target sites. These results prove that the CRISPR/Cas9 system is highly efficient and reliable for allotetraploid cotton genome editing.

## Introduction

Among the genome editing technologies, the prokaryotic‐traceable RNA‐guided Cas9 nuclease from type II clustered regularly interspaced short palindromic repeats (CRISPR) system takes the leading place because of its robustness and high efficiency, comparing to zinc finger nucleases (ZFNs) and transcription activator‐like effector nucleases (TALENs) (Gaj *et al*., 2013). The CRISPR/Cas9 system is primarily found in bacteria adaptive immunity against the invading bacteriophage and plasmids. It requires a crRNA and a tracRNA to form the two‐RNA structure, which is later integrated within one transcript and termed as sgRNA, to guide the Cas9 endonucleases to the target DNA sequences (Jinek *et al*., 2012). Generally, the crRNA is 20 bp in length and is complementary to the target site with a protospacer adjacent motif (PAM). This is the first explanation that how the CRISPR/Cas9 system works in mechanism. The guided Cas9 protein digests the target DNA site and generates a DNA double‐strand break (DSB) at a position about 3 bp upstream of the PAM sequence (Bhaya *et al*., 2011; Deltcheva *et al*., 2011; Horvath and Barrangou, 2010). The DSBs sites can be repaired by nonhomologous end joining (NHEJ) or homologous recombination (HR) with the former process introducing nucleotide deletions or insertions to destroy intact protein translation of the target gene (Symington and Gautier, 2011). These leading findings in prokaryote have provoked application of the CRISPR/Cas9 system in eukaryotic genome editing. The Cas9 from *Streptococcus pyogenes* was codon optimized on the basis of eukaryotic genomic characteristics. The tracRNA and crRNA were driven by species‐specific U6 RNA polymerase III (RNAPol III) promoters. Cong *et al*. (2013) successfully accomplished the first genome editing in human cells with CRISPR/Cas9 system. Simultaneously, Mali *et al*. (2013) conducted gene editing in different human cell types and accomplished DNA replacement with a donor template. Rice and wheat were the first crops that were genetically edited with CRISPR/Cas9 system, and knockout of *OsPDS* generated albino rice mutant (Shan *et al*., 2013). These pioneering works promote its broad utilization of CRISPR/Cas9 system in the research of life science.

Two separate vectors were used in the early applications, with one expressing the nuclear‐localized Cas9 endonuclease and the other transcribing sgRNAs driven by U6 promoter. To utilize this system, two vectors must be cotransformed into mamalian cells (Cong *et al*., 2013; Hwang *et al*., 2013; Mali *et al*., 2013), plant protoplasts and callus (Li *et al*., 2013; Miao *et al*., 2013; Shan *et al*., 2013), or infiltrated into leaves by Agrobacterium (Nekrasov *et al*., 2013). Moreover, for every single sgRNA, the RNAPol III promoter requires the start sites of the transcribed sgRNAs to specific ribonucleotide: GN_20_NGG for U6 promoter and AN_20_NGG for U3 promoter. Number of sgRNA targetable sites was also limited (Gao and Zhao, 2014; Sander and Joung, 2014; Xie *et al*., 2015). These limitations impeded the application of CRISPR/Cas9 system in generation of stable and heritable genome‐edited plants. Later, a single vector bearing both Cas9 and sgRNA expression cassettes was used for gene editing in mammalian cells (Ran *et al*., 2013). Besides, one vector method was also used for stable transformation in plants. In Arabidopsis and rice, a series of genes were tested for gene editing, and some transgenic plants showed phenotype as expected (Feng *et al*., 2013; Xie and Yang, 2013; Zhang *et al*., 2014). Now, it has been applied in other plants, including tomato, poplar, soya bean*,* petunia, marine algae and maize (Brooks *et al*., 2014; Char *et al*., 2016; Fan *et al*., 2015; Nymark *et al*., 2016; Sun *et al*., 2015; Zhang *et al*., 2016; Zhou *et al*., 2015).

Multiple sites targeting is also focused to achieve multiplex genes editing simultaneously. Primarily, two U6‐sgRNA‐terminator expression cascades were integrated within one vector (Li *et al*., 2013). Modifications were carried out to integrate series sgRNAs in one vector. A Gly transfer RNA (tRNA^Gly^) was used to release a series of sgRNAs from a single chimeric RNA in the processing of *in vivo* RNases P and RNases Z. This process exists in almost all organisms, and it accomplished multiple sites gene editing to a series of mitogen‐activated protein kinase (MAPK) genes in regenerated rice (Xie *et al*., 2015), which will broaden its application of CRISPR/Cas9 in lots of organisms, especially in polyploids for homologous gene mutation.

Cotton is an important economic crop because its fibre is an essential raw material in texile industry. The widely cultivated cotton species *Gossypium hirsutum* (A_1_D_1_) is an allotetraploid with a genome size of 2.5 Gb. Because of its homologous sequences and large portion of repeats, two draft genome maps were accomplished until 2015, with the reference genome information of two diploid progenitors, *Gossypium raimondii* (D_5_) and *Gossypium arboreum* (A_2_) (Li *et al*., 2014, 2015; Paterson *et al*., 2012; Yuan *et al*., 2015; Zhang *et al*., 2015). The decoded cotton genome information will promote functional genomic research. However, genetically modified cotton mutants are rare, and most functional genomic researches rely on RNA interference of the target genes. It somehow produces undesirable results because of gene redundancy or high similarity of homologous gene sequence. The booming CRISPR/Cas9 technology leads us to apply its use in cotton for genome editing in order to explore gene functions and/or improve agricultural traits.

The *DsRed2* protein (*Discosoma* red fluorescent protein2) was firstly isolated from reef corals (*Discosoma* sp.), and it has been applied in plant molecular biology as a reporter because of its distinct advantages over other report proteins (Jach *et al*., 2001; Wenck *et al*., 2003). Ectopic expression of *DsRed2* in soya bean generated red colour in somatic embryos and seeds under white light, which made it convenient to detect transformed genes if they were fused with the *DsRed2* (Nishizawa *et al*., 2006). *AtCLA1* is responsible for chloroplast development, and its mutant has an albino phenotype (Mandel *et al*., 1996). Manipulation of G*hCLA1*, a homologous gene to *AtCLA1*, generated an albino phenotype in cotton young leaves that was similar to *cla1* mutant (Gao *et al*., 2013; Li *et al*., 2016).

In this study, we successfully utilized the CRISPR/Cas9 system in allotetraploid cotton and accomplished multiple sites genome editing. The gene *DsRed2* was chosen as a target. Firstly, an *DsRed2*‐overexpressed transgenic cotton line, which had similar phenotype with that of soya bean in seeds (Nishizawa *et al*., 2006), was created, and it was used as a receptor line for *DsRed2‐*specific editing. The *DsRed2*‐edited T0 plants obtained authentic gene mutation, and they had no red fluorescence at an excitation wavelength of 530–550 nm. Besides, the mutation was genetically inheritable in T1 progenies, with same phenotype to wild‐type YZ1, both in seeds and seedlings. All the mutations were verified with Sanger sequencing as well. For another target gene *GhCLA1*, an average of 75% regenerated T0 plants showed an albino phenotype. Gene editing was verified with Sanger sequencing as well as barcode‐based high‐throughput sequencing, which was more simple and highly efficient than the former method. No off‐target effect was detected at the potential off‐target sites with application of high‐throughput sequencing method. All these results prove that the CRISPR/Cas9 system is highly efficient and reliable in cotton genome editing.

## Results

### Cloning of *pGhU6* promoter and vector modification for cotton transformation

The vector pRGEB32 was originally exploited for rice transformation, and the sgRNA transcription was driven by *pOsU3* from rice genome (Xie *et al*., 2015). Similar to U6, *pOsU3* is a RNAPol III promoter, and it can efficiently transcribe complete sgRNAs. Species‐specific endogenous U6 promoter was taken into consideration. Through homologous search in cotton genome with *Arabidopsis* snoRNA gene *AtU6‐26*, several candidate genes, termed *GhU6.1*,* GhU6.4*,* GhU6.7* and *GhU6.9*, were identified, and all of them had a conserved U6 snoRNA sequence with *AtU6‐26*. In the promoter region, they all contained a conserved U‐snoRNAs specific upstream sequence element (USE): RTCCCACATCG, and a TATA‐like box (Figure S1). These results verified that snoRNA U6 was highly conserved in nucleotide sequence among different plant species (Waibel and Filipowicz, 1990). A 1‐kb DNA sequence upstream the start site of the conserved U6 snoRNA was designated as a *pGhU6* promoter. As *pGhU6.9* showed the highest similarity to *pAtU6‐26*, it was finally chosen to modify the vector. Its original Bsa I site was mutated with one ‘G’ substitution to avoid more than two Bsa I sites in the final expression vectors (Appendix S1). Two vectors were finally constructed, namely pRGEB32‐GhU6.9 containing the *hpt* (Hygromycin B phosphotransferase) selection marker for a second gene transformation and pRGEB32‐GhU6.9‐NPT II for an endogenous gene editing (Figure S2).

### The CRISPR/Cas9 system induced gene knockout of an exogenously expressed reporter gene *DsRed2*

**Figure 1:**
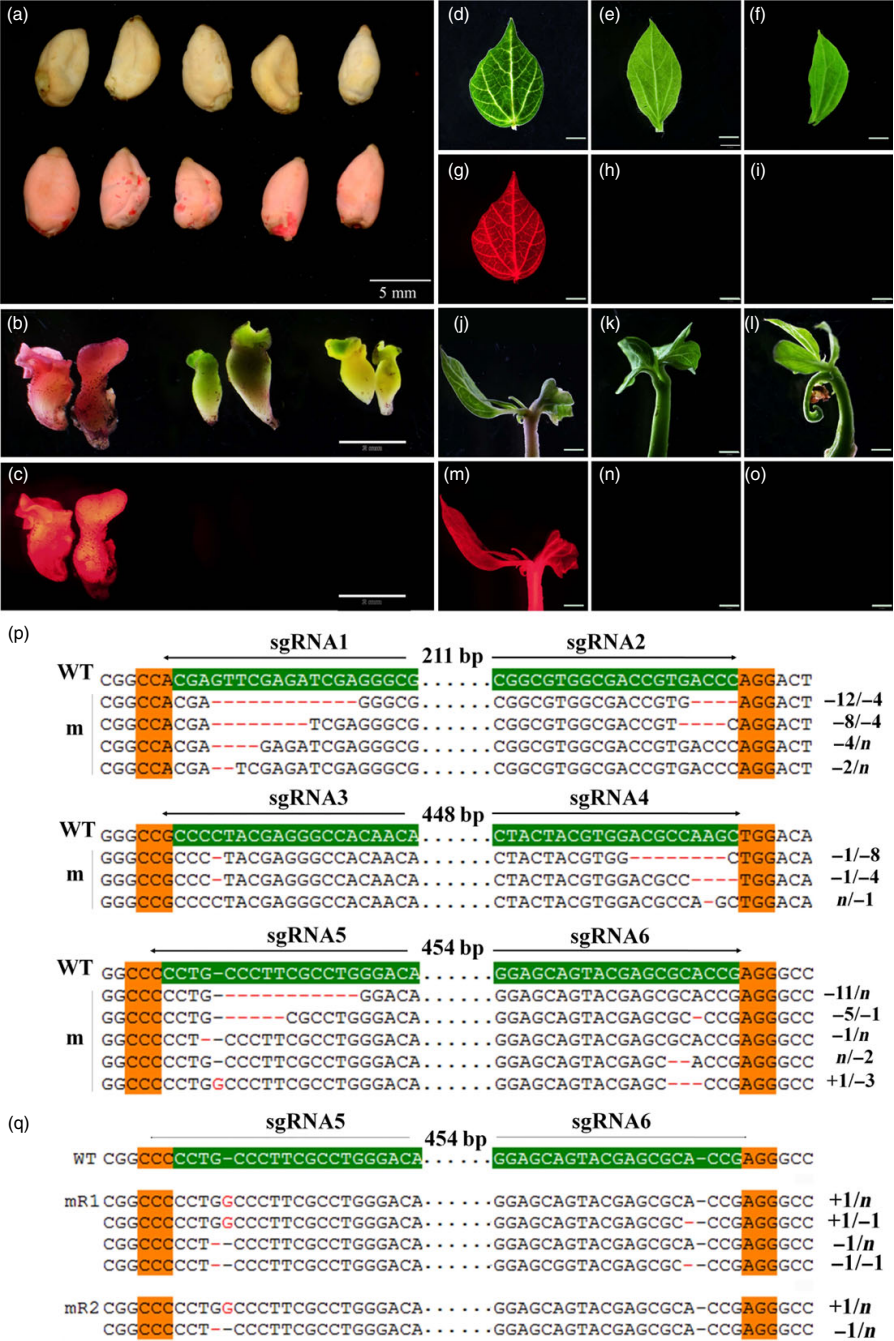
*DsRed2* mutation is induced by the CRISPR/Cas9 system. (a) Seeds of wild‐type cotton YZ1 (upper row) and a *DsRed2* overexpression line (bottom row). (b) and (c) Regenerated somatic embryos of the control line and two mutants (mR1 and mR2) in the white light field (b) and a red fluorescence field at an excitation wavelength of 530 to 550 nm (c). (d) to (o) Leaves and young seedlings from corresponding plants in (b) were observed in the white light field (d, e, f, j, k, l) and the red fluorescence field (g, h, i, m, n, o). Bar in (a) is 5 mm, in (b) to (o) is 2 mm. Sanger sequencing of somatic embryos (p) and two independent mutants (q) at the *DsRed2* target sites are exhibited. The sgRNA target sites are highlighted in green background. PAM regions are highlighted in orange. Nucleotide deletions or insertions are shown in red, with details labelled at right. The gaps between the paired sgRNAs are in dotted line, and their lengths are labelled above. WT, the wild type. m, mutation clones.

To test the modified pRGEB32‐GhU6.9 vector, the *DsRed2* was chosen as the target gene for editing. A *DsRed2* transgenic cotton line RED was previously created. All tissues of this line gained obvious red fluorescence at an excitation wavelength of 530–550 nm under a stereomicroscope (Figure S4), and its seeds were obviously in red colour (Figure 1a). This line was used as the receptor for a second *Agrobacterium*‐mediated genetic transformation. The vector pRGEB32‐GhU6.9 harbouring a *hpt* selection marker was chosen as the expression vector. Without integration of sgRNA, the empty vector was used as the control. Three pairs of sgRNAs were designed, thus getting three expression vectors containing polycistronic tRNA‐gRNA genes (*PTG*), *PTG1*,* PTG2* and *PTG3* (Figures S3 and S5a,c), and they were separately transformed into the receptor line with hygromycin screening.

It is well known that hygromycin is not frequently used in cotton transformation. In this study, we found that cotton explants were very sensitive to hygromycin and exhibited abnormal phenotype during regeneration. However, several regenerated lines with normal phenotype were obtained, and they were checked by PCR analysis with *Cas9‐* and *DsRed2*‐specific primers (Figure S6a). Three were *Cas9* positive. Under fluorescence stereomicroscope, red fluorescence was disappeared in both regenerated embryos and mature plants of *Cas9* positive lines, comparing to the control (Figure 1b–o). At differentiation stage, the embryos were sampled to check gene editing at the sgRNA target sites with Sanger sequencing. It demonstrated that gene editing, including nucleotide deletions and insertions, were observed at all the target sites (Figure 1p). Further sequencing of the *DsRed2* gene in two independent transgenic plants verified that mutations occurred in the gene coding region (Figure 1q), resulting in successful *DsRed2* knockout. This result confirmed that the modified vector with a cotton native promoter *pGhU6.9* has the capacity for genome editing in cotton.

### The CRISPR/Cas9 system induced *GhCLA1* gene mutation with an obvious albino phenotype in cotton

To verify the system's capacity for endogenous gene editing in cotton, *GhCLA1* was chosen as a target. Referring to cotton genome information, there were two *CLA1* copies belonging to At and Dt subgenomes, suggesting that four *CLA1* loci might be existed in cotton genome. Sequence alignment of *GhCLA1* gene copies showed that several SNPs are existed, which distinguish their origination from At or Dt subgenome (Figure S5b). Similar to *DsRed2* gene editing, two *PTG* genes, that is. *PTG4* containing sgRNA7‐sgRNA8 and *PTG5* containing sgRNA9‐sgRNA10, were finally generated (Figure S3 and S5c). The empty vector pRGEB32‐GhU6.9‐NPT II was used as the control during *Agrobacterium*‐mediated genetic transformation. These sgRNAs were designed for targeting both copies of *GhCLA1* in At and Dt subgenomes, except for sgRNA9. The sgRNA9 site contains a SNP at 3′ end of the target sequence, and it matches well with gene locus from D‐subgenome (Figure S5b). This SNP is just 2 bp upstream of the corresponding PAM motif.

**Figure 2:**
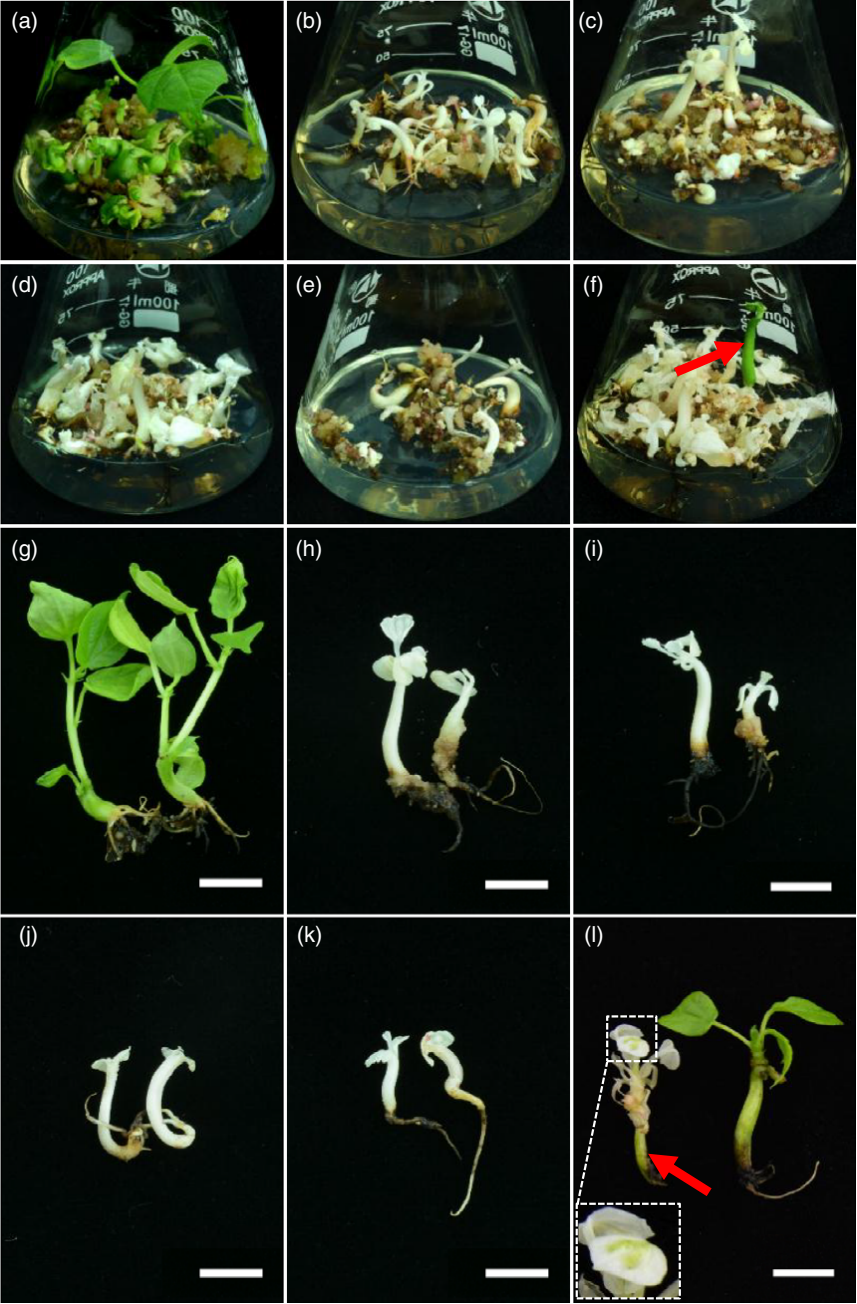
Genome editing of *GhCLA1* generates albino cotton plants. Regenerated somatic embryos of the control line (a) and five mutants (b–f) are shown. A green seedling among the albino plantlets is pointed out with a red arrow (f). Young plantlets of the corresponding control line (g), four mutants (h–k) and mosaic and green ‘mutants’ (l) are exhibited. Green part of the mosaic plant is highlighted in a magnified dash box and a red arrow (l). Bars in (g) to (l) are 1 cm.

**Figure 3:**
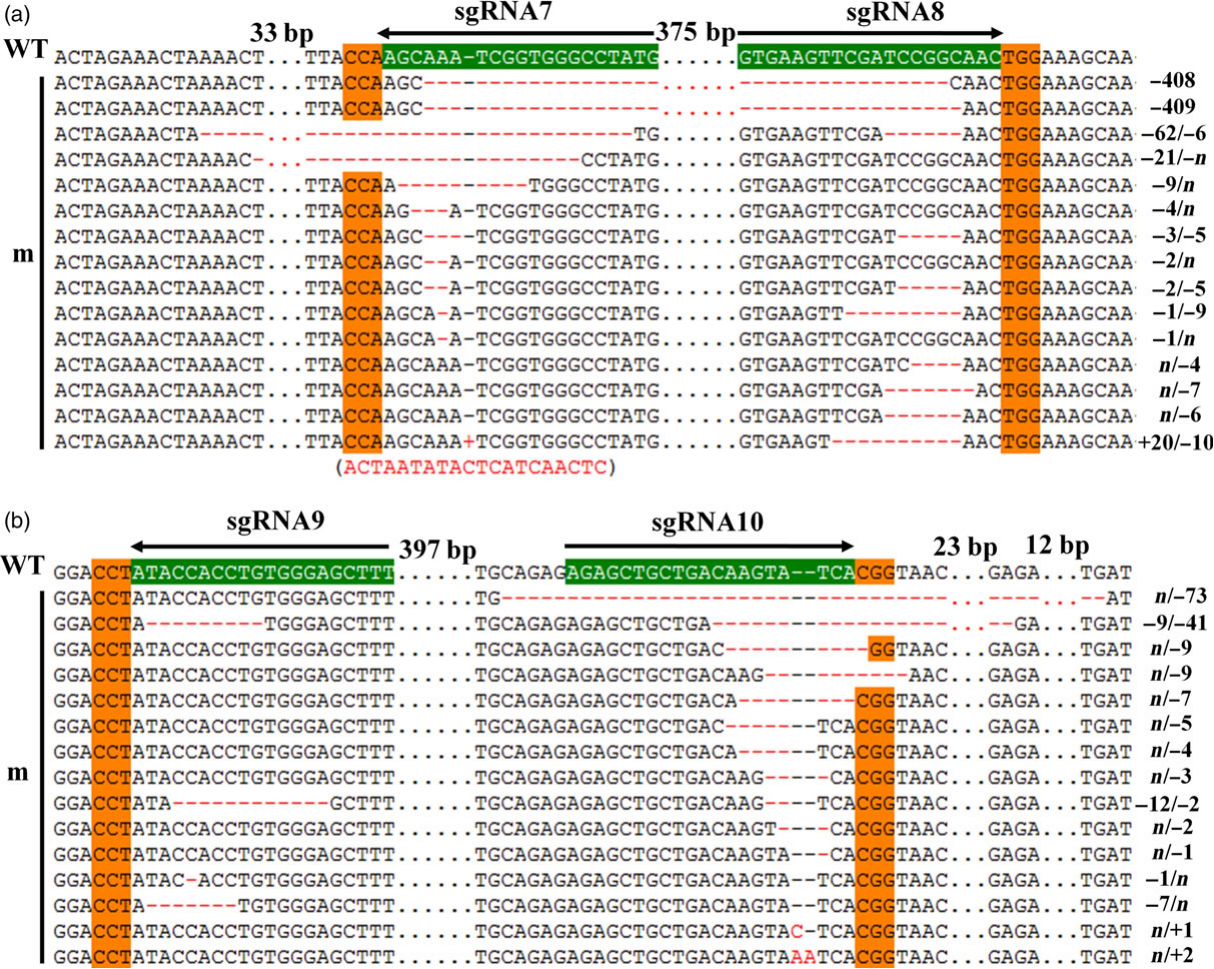
Sanger sequencing of somatic embryos at *GhCLA1* target sites. (a) Genome editing at sgRNA7 and sgRNA8 sites. (b) Genome editing at sgRNA9 and sgRNA10 sites. The sgRNA target sites and the PAM regions are highlighted in green and orange background, respectively. Nucleotide deletions or insertions are shown in red, with details labelled at right. The gaps between the omitted nucleotides are in dotted line, and their lengths are labelled above. A 20 bp insertion is shown in brackets under the sequence. WT, the wild type. m, mutated clones.

At the differentiation stage, somatic embryos/plantlets exhibited albino as compared to the control (Figure 2a–f). To further investigate detailed gene editing at the sgRNA target sites, gene region of *CLA1* was amplified from genomic DNA of embryos, and Sanger sequencing was conducted. The results demonstrated that gene editing was observed at all the target sites (Figure 3). For every single target site, nucleotide deletions were most abundant, ranging from 1 to 73 bp. As predicted, large fragment deletions of 408 and 409 bp were observed between sgRNA7 and sgRNA8 target sites. An insertion of 20 bp nucleotides was also detected at sgRNA7 site (Figure 3). More importantly, many plantlets exhibited an albino phenotype and obvious growth retardation, whereas the control plants had green leaves and exhibited normal growth (Figure 2g–l). Plants with a chimeric phenotype have been shown in Figure 2l. Interestingly, a single green seedling was found among the albino plants that were generated from the same cell line (Figure 2f). Finally, 13 and 15 independent T0 transgenic plants for pPTG4 and pPTG5 vectors, respectively, were generated and analysed in this report. All these plantlets were checked with *Cas9*‐specific primers and *CLA1* gene primers (Figure S6b).

**Figure 4:**
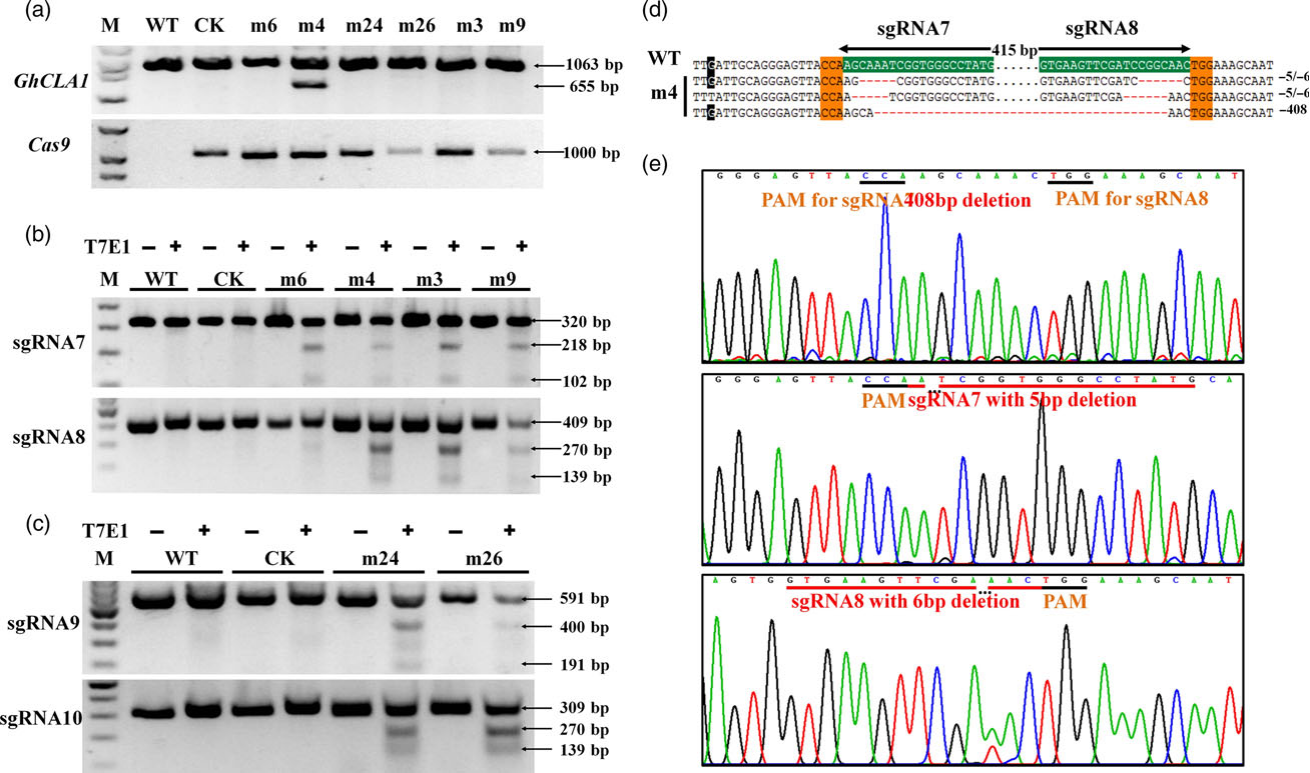
Mutation at *GhCLA1* target sites with T7E1 digestion assay and Sanger sequencing. (a) *GhCLA1* and *Cas9* positivity check of the wild type (WT), control (CK) and mutants. Mutants m3 and m9 are in chimeric and green phenotype. Mutants m6, m4, m3 and m9 were targeted at sgRNA7 and sgRNA8 sites. Mutants m24 and m26 were targeted at sgRNA9 and sgRNA10 sites. (b) and (c) T7E1 digestion assay of the mutants at the target sites. M, marker. A large fragment deletion was occurred in a representative albino mutant m4. Its Sanger sequencing results (d) and the detailed chromatograms (e) at the target sites are illustrated. In (d), the SNP is highlighted in black. In (e), the top chart illustrates the large fragment deletion of one *GhCLA1* copy. The charts at middle and bottom represent mutations at separate target sites of the same gene locus. The PAM regions and mutated target sites are underlined with black and red lines, respectively.

T7E1 enzyme digestion assay was conducted to check *GhCLA1* mutation. For each independent plant, the two sgRNA sites were analysed separately. The results showed that except for the wild type and control plants, other mutants generated two shorter fragments, including the chimeric and green plants (Figure 4b,c). Summation of these two fragments size was equal to the target band that was shared in all plants. It illustrates that mutations indeed occurred at these target sites. Noteworthy, the mutant m4 showed an extra band that was approximately 400 bp smaller than the target *CLA1* fragment (Figure 4a). Tracing back to embryonic callus stage, this plant was originated from the same transgenic cell line which had a 408‐bp fragment deletion between the sgRNA7 and sgRNA8 target sites. The mutation was verified with Sanger sequencing as shown in Figure 4d,e. It clearly showed that there were three types of editing at all the *CLA1* loci. Gene editing from At and Dt subgenomes could be distinguished by the SNP. This result confirmed our prediction that using a pair of sgRNAs can generate large fragment deletion, which would be helpful to study the function of large cisregulatory domains or gene clusters (Zhou *et al*., 2014).

### The albino phenotype was generated by homozygous mutation at all *GhCLA1* loci

For most albino plants, they were white in uniformity. Whereas a few of chimeric or green seedlings appeared among the pale plants (Figure 2f). It impelled us to investigate the exact reason why they exhibited differently. Further, Sanger sequencing for independent T0 plants was conducted. The genomic *CLA1* DNA fragment that covers all the designed sgRNAs target sites was the target sequence. Sanger sequencing results were obtained for all these regenerated 28 plants. These sequencing results were classified into two groups belonging to At or Dt subgenome, respectively, according to the SNPs information in *GhCLA1* genome sequence (Figure S5b). Summary of plants phenotype and mutation genotype is listed in Table 1. The results demonstrated that gene editing occurred more easily than the predicted phenotype. For example, three plants from independent lines, m9, m10 and m11, were edited at each sgRNA target site, but they still exhibited green phenotype (Table 1). This was due to mono‐allelic mutation at each target site. However, in the albino plants, bi‐allelic mutation was occurred at all the loci in At and Dt subgenomes at either one of the target sites. Thus, we speculated that only mutations occurred in both target sites of At and Dt subgenomes simultaneously could generate a complete pale phenotype.

**Table 1:** Mutation genotype and phenotype of independent *GhCLA1*‐edited T0 plants

Line	Phenotype	Cas9 positivity	Mutation at sgRNA7 site		Mutation at sgRNA8 site	
			At	Dt	At	Dt
m1	White	Y	−2, n	−2, n	+1, −3, −4	−4, −5
m2	White	Y	−3	−2, n	−5	−3, −4
m3	Chimeric	Y	n	−1, −2, −5, n	−3, −4, n	+1, −5, −7, 10, 11
m4	White	Y	−5	−408, −5	−6, −6	−6, −408
m5	White	Y	+1, −2	−4, −6	−22, −5	−4, +1
m6	White	Y	−2, −19	−3	−6, −6	−5, −7
m7	Chimeric	Y	−2, n	+1, −3, −4, n	−4, n	+1, −5
m8	White	Y	−1, −2	−3	−1, −7, −43	−6, −6
m9	Green	Y	−4, −5, −7, n	−11, n	−3, n	−2, n
m10	Green	Y	n	n	−6, n	−4, n
m11	Green	Y	n	−2, n	−5, n	−4, n
m12	White	Y	−1, n	−3, −5, n	−5, −5	−5, −4
m13	White	Y	−1, −5	−2, n	−4, −6	−7, −7

Line	Phenotype	Cas9 positivity	Mutation at sgRNA9 site		Mutation at sgRNA10 site	
			At	Dt	At	Dt
m21	White	Y	n	n	−3	−2
m22	White	Y	n	n	−3, −13	−7
m23	Green	Y	n	−4, n	n	−7, n
m24	White	Y	n	−3, −19	−2, −8	−7, −8
m25	White	Y	n	−3, −4, n	−12	−2, −7
m26	White	Y	n	−2, −3, −6	+1, −2	−2, −24(+19)
m27	White	Y	n	−4, −7	−1, −7	−1, −7
m28	White	Y	n	n	−2, −7	−1, −4, −6
m29	White	Y	n	−1, n	−17	−3, −5, −20
m30	White	Y	n	−3, −9	−7, −10	+10, −1, −50
m31	Green	Y	n	−3	−1, n	−2
m32	White	Y	n	n	−1, −2	−2, −6
m33	White	Y	n	−5, −19	−7, −8	−8, −35
m34	White	Y	n	−1	−3, −5	−2, −60
m35	White	Y	n	n	−7	−1

‘−’, ’+’ and ‘n’ represents nucleotide deletions, insertions and no mutations at the target sites, respectively. The number in bracket means newly inserted nucleotides at the deleted site.

Detailed analysis of each target site showed that the vector harbouring sgRNA10 as guide RNA had the highest homozygous mutation efficiency, with a ratio of 80.0% (Table 2). However, at the paired sgRNA9 target site, gene editing was only observed at *GhCLA1* loci from Dt subgenome. No target loci from At subgenome were edited at all. It was the mismatched single nucleotide at the target site of At subgenome that made such a difference. Moreover, this mismatch site is located at the second nucleotide upstream of the PAM region. This result verified that the CRISPR/Cas9 system was more sensitive to mismatches in the PAM‐proximal regions than to PAM‐distal regions (Kuscu *et al*., 2014). For the other two target sites, sgRNA7 and sgRNA8 had a ratio of homozygous mutation with 30.77% and 61.54%, respectively. Furthermore, the total editing efficiencies in *GhCLA1* loci (i.e. At or Dt subgenome or AtDt homozygous editing) for sgRNA7, sgRNA8, sgRNA9 and sgRNA10 were 92.31%, 100%, 66.7% and 100%, respectively (Table 2). Considering the phenotype, 61.54% of transgenic plants harbouring pPTG4 vector were albino. The remaining transformants were chimeric and green, with a ratio of 15.38% and 23.08%, respectively. The corresponding ratios were 86.7%, 0%, 13.3% for pPTG5 vector, respectively. In summary, the average ratio of plants with predicted albino phenotype was up to 75% among the regenerated plants, confirming that our CRISPR system had a very high genome editing efficiency in cotton.

**Table 2:** Mutation rates of *GhCLA1* at each target site and phenotype

*PTG* gene	Target site	Mutation rate at each site, %	Homologous mutation rate at each site, %	Mutation rate at both sites, %	Mutation rate of phenotypes		
					Albino, %	Chimeric, %	Green, %
*PTG4*	sgRNA7	92.31 (12/13)	30.77 (4/13)	92.31 (12/13)	61.54 (8/13)	15.38 (2/13)	23.08 (3/13)
	sgRNA8	100 (13/13)	61.54 (8/13)				
*PTG5*	sgRNA9	66.7 (10/15)	0 (0/15)	66.67 (10/15)	86.7 (13/15)	0 (0/15)	13.3 (2/15)
	sgRNA10	100 (15/15)	80.0 (12/15)				
Total	–	–	–	–	75.0 (21/28)	7.14 (2/28)	17.86 (5/28)

### Gene mutation at the target sites was genetically inheritable to T1 progenies

**Figure 5:**
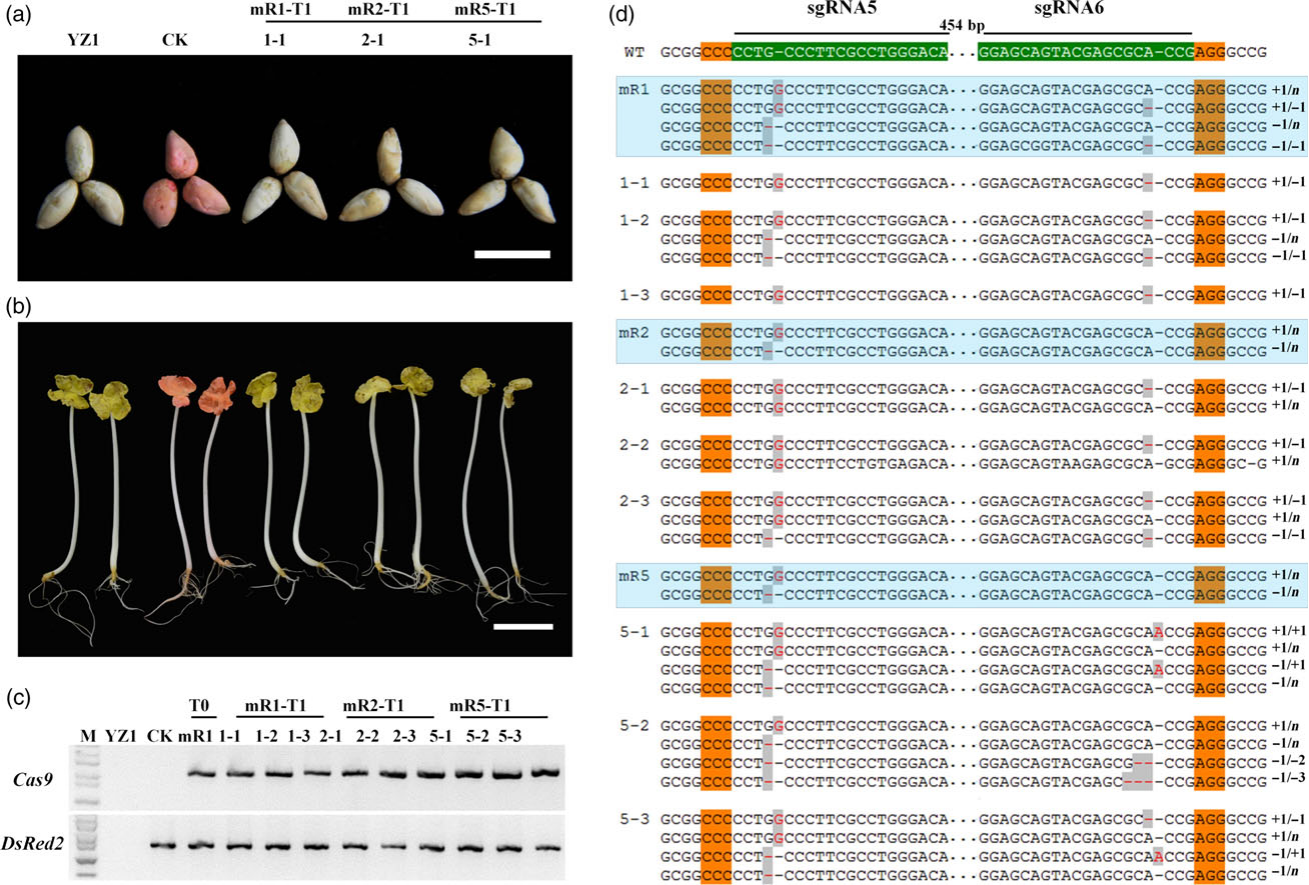
*DsRed2* mutation is genetically inheritable from T0 to T1 generation. Seeds (a) and seedlings (b) of YZ1, the control and three representative T1 plants. (c) PCR analysis of *Cas9* and *DsRed2* in YZ1, CK, the T0 mutant mR1 and T1 progenies. (d) Genotyping of the target *DsRed2* gene of independent T0 plants and their T1 progenies. Results for the T0 plants are highlighted in blue textbox. Mutated sites are highlighted in red with grey background. Bars in (a) and (b) are 2 and 3 cm, respectively.

Above results verified that the CRISPR/Cas9 system did work in regenerated T0 cotton plants. Further, we checked the genetic inheritance of *DsRed2* mutation in T1 progeny. For *GhCLA1*, the T0 plants ceased growth and could not produce seeds as normal adolescent plants. So only the progenies generated from the mR1, mR2 and mR5 mutants of *DsRed2* were analysed in this report. The T1 seeds and seedlings exhibited the same phenotype (no red colour) as the wild type. Whereas, the control plant without genome editing still showed red colour in both seeds and whole seedlings (Figure 5a,b). All the plants were checked with *Cas9‐* and *DsRed2*‐specific primers, confirming they were transgenically positive (Figure 5c). Southern blotting revealed that the three T0 mutants had four *DsRed2* copies. Their progenies were obviously segregated from these T0 plants (Figure S7). At the sgRNA5 site, the mutation genotype in T0 was one inserted ‘G’ and one deleted ‘G’ in all the T0 plants. But at the sgRNA6 site, there was no editing in mR2 and mR5 (Figure 5d). In T1 generation, the plants kept at least one kind of mutation at sgRNA5 target site. The segregation of mutation genotype was in accordance with the Southern results (Figure S7). Interestingly, at the sgRNA6 target site, the T1 progenies of mR2 gained new mutations with one ‘A’ deletion. For the mR5 progenies, one ‘A’ insertion and two or three nucleotide deletions were observed (Figure 5d). These new mutations might occur during reproduction or development stages of the T1 progenies. All these results proved that gene mutation generated with CRISPR/Cas9 was inheritable.

### Barcode‐based high‐throughput sequencing was used to detect *GhCLA1*‐edited plants with high efficiency

The above case for *GhCLA1* editing illustrated high potential of CRISPR/Cas9 in cotton. Nevertheless, genome editing of *GhCLA1* generated an obvious phenotype which was easily recognized at early stages for transgenic plants. For most genes, their mutants might not have distinguishable phenotypes at seedling stage, especially for those participating in biotic and abiotic responses, or in fibre development. Moreover, a homologous mutant should have all the gene loci, at least two for each gene, be edited in cotton. Detection of the target sites of each plant with Sanger sequencing was time‐consuming and costly. A better method should be applied for mutants screening, especially when a large population of mutants were generated.

**Figure 6:**
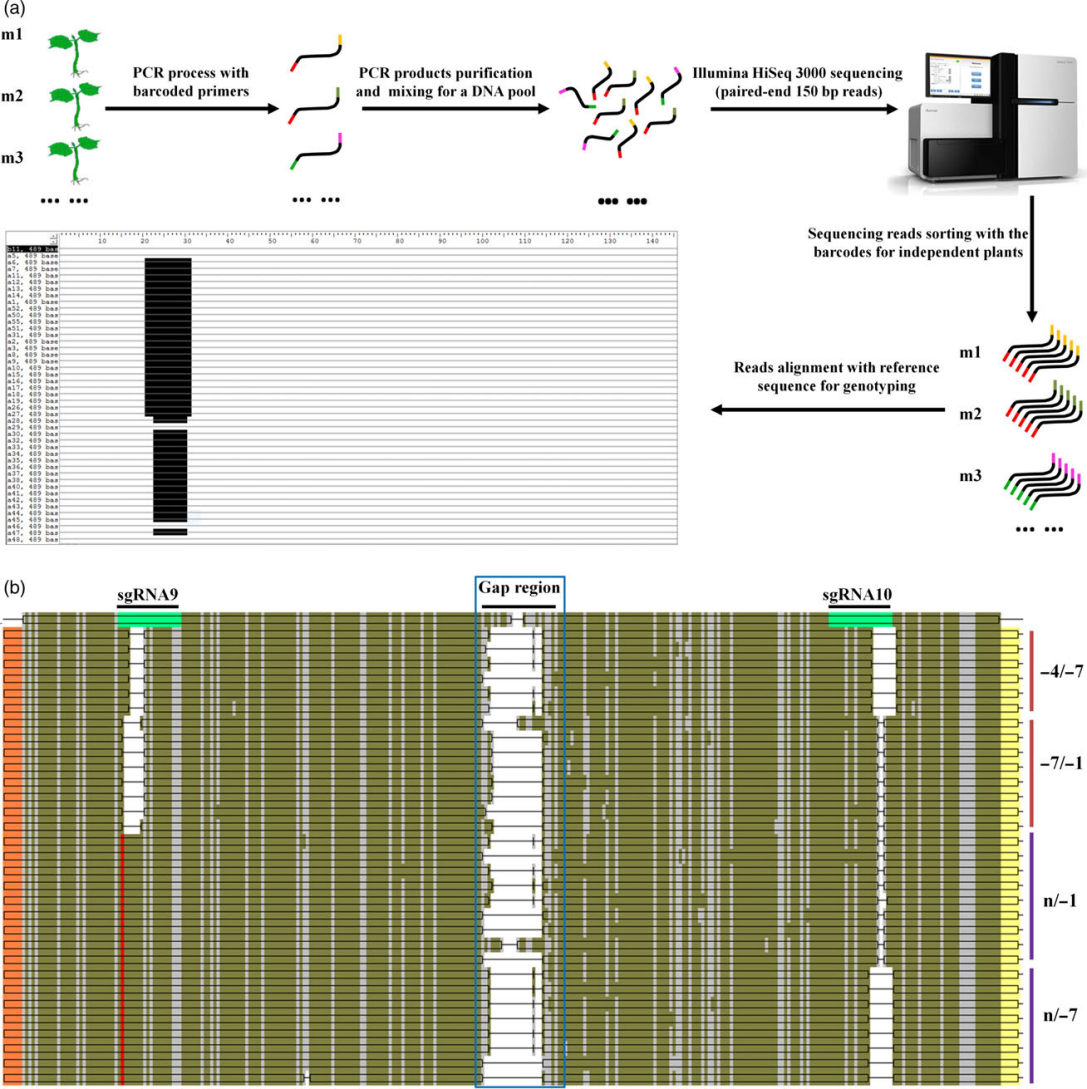
Barcode‐based high‐throughput sequencing is applicable to detect a large population of mutants in a run. (a) Pipeline of the barcode‐based sequencing. The barcodes (coloured) are added to 5′ end of the primer. (b) A representative alignment of sequencing reads from *GhCLA1*‐edited mutant m27. The sgRNA target sites in the reference DNA are highlighted in green. The barcodes in sequencing reads are highlighted in orange and yellow. The regions in blue box represent the sequencing gap for the paired reads. The SNP against the sgRNA9 site is illustrated in red. Nucleotide deletions at the target sites are in blank, with details labelled at right. The brown and purple lines represent the origination of the reads from D and A subgenome, respectively.

Barcode strategy has been used in single cell omics for tracing DNA or RNA that is originated from separate cells (Rotem *et al*., 2015; Satija *et al*., 2015). This application allows high‐throughput sequencing of combined cells, or multiple gene loci in one library, and the data can be sorted by the designed barcodes for every independent target. To test its practicability in cotton, we designed a series of barcodes at 5′ ends of *GhCLA1*‐specific primers (Table S1). Namely, all the 28 plants had a pair of specific barcodes to mark them. The barcoded PCR products from independent transgenic plants were obtained with corresponding primers. They covered the paired sgRNA target sites for independent plants. The products were purified and mixed with equal amount to build one DNA library that was consequently applied to Illumina HiSeq 3000 system for paired‐end 150 bp reads (Figure 6a). Finally, a total of 137 545 unique reads were obtained, and they were sorted with the specific barcoded primer pairs for corresponding plants. The total unique reads for all the plants were 10 845 (Table S2). The sorted reads of each plant matched well with *GhCLA1* genome sequence. Mutations at the target sites were detected, and they were comparable with the results of Sanger sequencing (Figure 6b). To summarize, it was practical to use the barcodes to label the target DNA products from independent transgenic lines and to apply high‐throughput sequencing to identify a large population of mutants.

### No off‐target effect was detected in albino plants using a high‐throughput sequencing method

Even though the successful gene editing of *GhCLA1* was accomplished, we were still concerned about unpredicted mutations in nontarget gene regions. For all the sgRNA target sites, we searched their highly potential off‐target sites according to Xie *et al*. (2014). These potential off‐target sites and their related genome positions were listed in Table 3. There were 6, 4, 7 and 9 sites for sgRNA7, sgRNA8, sgRNA9 and sgRNA10, respectively. Among them, three sites locate in the exon region of four protein coding genes, which are represented with gene ID. The others locate in noncoding regions. These sites were searched with canonical 5′‐NGG PAM standard. Four sites with 5′‐NAG PAMs were also considered because it was previously reported that Cas9 also recognizes this kind of PAMs to cleave target sites (Hsu *et al*., 2013). A recent study demonstrates that sgRNA target sites with noncanonical NGG PAMs, including NAG, NGG and NGA, also have notable sgRNA activity (Doench *et al*., 2016). A 300 bp DNA sequence that covered each site was used for primer design (Table S4, Appendix S2). Mixed genomic DNA from the mutated transgenic plants was used as the template. A total of 26 PCR products were obtained and mixed with equal amount in one library for high‐throughput sequencing (Illumina HiSeq 3000, paired‐end 150 reads).

**Table 3:**
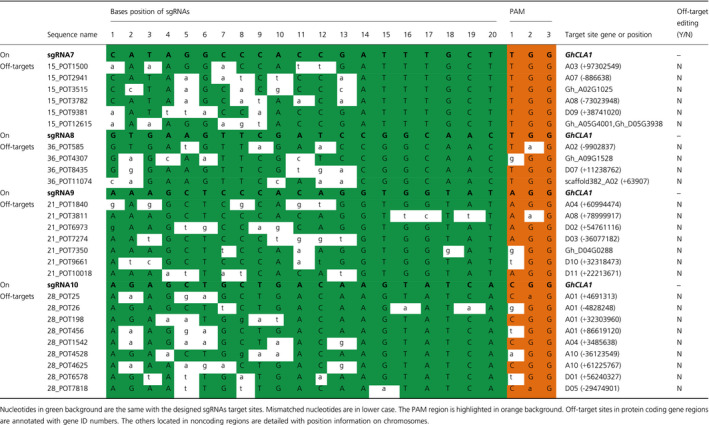
Detection of potential off‐target effect for each sgRNA target site

Finally, a total of 337 594 unique reads were obtained, and they were sorted with the primer pairs for each potential off‐target sites, namely 26 groups in total (Table S3). Reads from each group were aligned with the reference DNA sequences to detect mutations. Most reads matched well with the reference DNA, with a few of reads having SNPs at the off‐target sites but they were not edited. Besides, unexpected reads that did not match well with the reference DNA were found. That might be due to primer mismatches during PCR process (Figure S8). The final results showed that there was no editing at all the potential off‐target sites (Table 3), implying that our system had a high specificity in target gene editing in cotton.

## Discussion

Since its successful application in human cells, the CRISPR/Cas9 system continually brings a revolutionary trend in genome editing both in prokaryotes and eukaryotes. Its precise editing in the target gene can generate mutants. Development of this system made it possible to achieve high‐throughput screening for functional gene research at genome level (Wang *et al*., 2016; Zhou *et al*., 2014). Further research modified Cas9 to induce gene knockdown or transcriptional activation (Gilbert *et al*., 2014; Konermann *et al*., 2015). Till now, its utilization has been focused on noncoding transcriptional regulatory elements to elucidate the function of enhancers (Lopes *et al*., 2016). The improvements of this system also facilitated its utilization in plants, especially for crops modification, including rice, wheat, soya bean and maize (Char *et al*., 2016; Jacobs *et al*., 2015; Shan *et al*., 2014; Wang *et al*., 2014). Our successful application of this system in cotton will broaden its utilization of CRISPR/Cas9 system in plant kingdom, especially for polyploidy species.

In this study, we successfully mutated an endogenous gene *GhCLA1* and an exogenous gene *DsRed2* with the CRISPR/Cas9 system (Figures 1 and 3). The vectors used in this report could assemble several sgRNAs in one vector for multiple sites editing, with a tRNA pattern to separate each sgRNA cascade. The chimeric multiple sgRNAs could target many sites at the same time. Two sgRNAs were assembled for one target gene on the purpose of efficient mutation by large fragment deletion. It finally worked, with high efficient deletions from 1 bp to large fragment of 408 bp. Interestingly, there were more than two types of mutation at one gene copy in some albino plants. For example, in m28 line, the sgRNA10 target sites in Dt subgenome had three types of gene mutation. At some target sites, four mutation genotypes were observed (Table 1). This might be because of cell inhomogeneity from the regenerated plants. It was very obvious in chimeric plants, the leaves of which exhibited albino and dotted green phenotype (Figure 2l).

*GhCLA1* has two copies that belong to At and Dt subgenomes, respectively. SNPs are distributed along the exons and introns regions. Within the target site regions, 12 SNPs existed (Figure S5b), which helped us to distinguish the origin of these gene copies. Analysis of the mutation genotype using SNP information demonstrated that only di‐allelic mutation of all the homologues gene copies could generate albino mutants (Table 1). Those green or chimeric plants were partially mutated at some gene loci. This is similar in polyploid‐like hexaploidy bread wheat (Wang *et al*., 2014). Therefore, before sgRNA design, we should search the plant genome and make it clear how many copies of the target gene are existed in the genome. Moreover, SNPs among homologous genes or alleles helped us to design sgRNAs for mutation of specific gene loci. Our successful application in this study provides insights for precise gene function analysis to specific gene copies or alleles.

The inheritance of target gene mutation is a main focus to utilize CRISPR/Cas9 system. Identical mutations of *DsRed2* were detected in T1 generation, and it verified that the mutation was inheritable (Figure 5). It was stable both in genotype and phenotype from T0 to T1 generation. Noteworthy, new mutations, which were failed in T0 plants, were generated in T1 progenies at the target sites. This confirms that the CRISPR/Cas9 system has sustaining ability to conduct gene editing as long as the target site is wild type (Zhang *et al*., 2014). Unfortunately, we failed to screen out transgene‐free plants with authentic mutation because of the limited population size of T1 progenies. It is urgently needed to commercialize transgene‐free crops that are genetically engineered with gene transformation methods. CRISPR/Cas9 system has been applied to Arabidopsis, tobacco, lettuce, rice and wheat to generate transgene‐free plants (Woo *et al*., 2015; Zhang *et al*., 2016). This system would be more applicable and flexible for crop modification.

Successful genome editing of *GhCLA1* and *DsRed2* encourages us to broadly apply the CRISPR/Cas9 system in cotton. Nevertheless, not all T0 plantlets will exhibit obvious phenotype at seedling stage such as *GhCLA1* and *DsRed2* genes, especially for those genes functioning in stress resistance or plant development at later stages. It would be quite essential to identify homologous mutants at T0 stage quickly and precisely with the help of sequencing. Sanger sequencing is reliable but it would be time‐consuming and costly, thus constrains the efficiency to conduct high‐throughput applications. The barcode‐based high‐throughput sequencing has been applied for genotyping for lots of target genes in zebrafish (Varshney *et al*., 2016). In this study, the barcodes strategy was combined with next‐generation sequencing methods, and it achieved mutation genotyping for dozens of mutants in a run. Similarly, high potential off‐target sites were also checked with high‐throughput sequencing method with one mixed DNA library. It was exciting that there was no off‐targeting in the 26 sites, implying that the CRISPR/Cas9 system is highly specific in cotton. This is the same as that in wheat (Zhang *et al*., 2016). It seems that off‐targeting happens more easily with high frequencies in human cells (Fu *et al*., 2013; Pattanayak *et al*., 2013) than that in plants, such as rice and soya beans (Jacobs *et al*., 2015; Xu *et al*., 2015). Therefore, the next‐generation sequencing method will be practical to detect mutations of independent plants simultaneously and efficiently. It will extend our research to high throughput.

The high efficiency of CRISPR/Cas9 system in cotton prompts us to further exert its great potential for functional genomic research. In the future, modifications will continue to broaden its use from mutant generation to precise gene regulation at noncoding enhancer regions. These applications will not be restricted to single gene locus. A series of target genes in the same metabolic or signalling pathway can be manipulated simultaneously. In summary, CRISPR/Cas9 system still has a wide prospect of application in diverse plant species, especially for polyploid crops with complex genomes, such as wheat, oilseed rape and cotton.

## Experimental procedures

### Vector modification for cotton genetic transformation

Four promoter sequences, termed *pGhU6.1*,* pGhU6.4*,* pGhU6.7* and *pGhU6.9*, were amplified from cotton genome and verified with Sanger sequencing. Promoter *pGhU6.9* was finally chosen for vector modification because of its high identity with that of *AtU6‐26* (Figure S1). Its Bsa I restriction site was mutated to avoid multiple Bsa I sites in the final vector (Appendix S1). The pRGEB32 plasmid, a gift from Xie *et al*. (2015), was linearized with Hind III and Sbf I double digestion, resulting in deletion of gRNA‐terminator fragment. The promoter *pGhU6.9* was assembled with the gRNA‐terminator segment using an overlapping PCR method. The assembled fragment was inserted to the linearized pRGEB32 using ClonExpress® II One Step Cloning Kit (Vazyme, Nanjing, China), thus generating pRGEB32‐GhU6.9 vector. It has a *hpt* selection marker and was used to target a reporter gene *DsRed2*, which was firstly transformed into cotton with a vector carrying *NPT II* (Neomycin phosphotransferase *II*). For endogenous gene targeting, we changed the selection marker *hpt* with *NPT II* between two restriction sites, Pspx I and Xmal I, using above‐mentioned one step cloning strategy (Figure S2). This vector, defined as pRGEB32‐GhU6.9‐NPT II, was used for stable genetic transformation to knock out *GhCLA1* in cotton.

### Expression vector construction

For *DsRed2* gene editing, six sgRNAs were designed in the coding region, namely sgRNA1 to sgRNA6. Every two sites were designed to be integrated in a single vector. The pGTR plasmid, a gift from Xie *et al*. (2015) was used to amplify tRNA^Gly^ and gRNA fragments. Fragments containing tRNA‐sgRNA1 fusion and gRNA‐tRNA‐sgRNA2 fusion were obtained using pGTR as template. These two fragments were fused together with an overlapping extension PCR, thus getting gene *PTG1*. It was finally ligated to Bsa I‐digested pRGEB32‐GhU6.9 vector using ClonExpress® II One Step Cloning Kit (Vazyme) to obtain pPTG1 construction (Figure S3). Similarly, another two pPTG vectors were constructed, with pPTG2 containing sgRNA3 and sgRNA4 combination and pPTG3 containing sgRNA5 and sgRNA6 combination. For *GhCLA1* gene editing, four sgRNAs were designed in the exon region, namely sgRNA7, sgRNA8, sgRNA9 and sgRNA10. Two *PTG* genes, *PTG4* containing sgRNA7‐sgRNA8 pair and *PTG5* containing sgRNA9‐sgRNA10 pair, were generated in the same procedure for *PTG1* construction. pRGEB32‐GhU6.9‐NPT II was used as an expression vector, with *PTG4* and *PTG5* inserted into the Bsa I site to obtain pPTG4 and pPTG5 expression vectors. The expression vector pCAMBIA2300 harbouring the *Discosoma red fluorescent protein 2* (*DsRed2*) gene was obtained from Biotechnology Center of DBN company of Beijing as a gift. All the constructed pPTG plasmids, pPTG1 to pPTG5, and pCAMBIA2300‐*DsRed2* overexpression vector were separately transformed into *Agrobacterium tumefaciens* strain EHA105 for cotton transformation. The primers used in vector construction are listed in Table S4.

### *Agrobacterium*‐mediated transformation of cotton

Cotton cultivar *G. hirsutum* cv. YZ1 was used as the transformation receptor for *DsRed2* overexpression and *GhCLA1* gene editing in this study. A *DsRed2* overexpression line was used as the receptor for a second gene transformation to knock out *DsRed2*. Seeds of the receptor plants were sterilized and cultured in a chamber without light for 6 days at 30 °C. Their hypocotyl was used as explants for *Agrobacterium*‐mediated transformation following our previous reports (Jin *et al*., 2006a,b; Tian *et al*., 2015). All the regenerated plants were grown in a tissue culture room with a 14 h light/10 h dark condition at 25 °C.

### Fluorescence microscopy imaging

Somatic embryos and young leaves of *DsRed2*‐edited cotton were observed with an Olympus stereomicroscope SZX16 (Olympus, Tokyo, Japan). The red fluorescence was observed at an emission wavelength of 575 nm with an excitation wavelength of 530–550 nm.

### DNA extraction, PCR verification, Southern blotting and Sanger sequencing

Genomic DNA of the cotton plants was extracted with Plant Genome Extraction Kit (TIANGEN, Beijing, China). For transgenic positivity check, specific primers of *Cas9* sequence were used in PCR analysis. Southern blotting was conducted with *NPT II* fragment as the DNA probe to detect *DsRed2* copies using DIG High Prime DNA Labelling and Detection Starter Kit II (Roche, Basel, Switzerland). Partial *GhCLA1* sequences that covered sgRNA7‐sgRNA8 sites and sgRNA9‐sgRNA10 sites, respectively, were used for mutation genotyping in independent transgenic lines. The obtained PCR products were ligated in pGEMT‐Easy vector for TA cloning with T4 DNA ligase (Promega, Madison, WI). After reaction, the ligated products were transformed into *Escherichia coli* strain Top10, and positive clones were applied for DNA Sanger sequencing.

### T7E1 assay for mutation check

Genome DNA that covered each sgRNA site was amplified from independent lines. Primers used were listed in Table S4. About 500 ng PCR products of each sample was used to detect mutation with T7 Endonuclease I (NEB, Ipswich, MA) according to the manufacture's instruction. Final reaction products were analysed with 1.5% agarose gel electrophoresis.

### Barcodes design and high‐throughput sequencing

A DNA barcode with six nucleotides was added to 5′ end of *GhCLA1*‐specific primer pairs. A total of 13 barcodes were designed, and they were checked with the *GhCLA1* DNA sequence to avoid mismatch during PCR amplification. Finally, 30 nonrepetitive combinations, with forward and reverse as a pair, were chosen for corresponding plants (Table S1). The wild‐type DNA was also included as a control. To cover sgRNA7 and sgRNA8 target sites, a 501 bp PCR product was used, and it was 508 bp for sgRNA9 and sgRNA10 pairs. Genomic DNA from the corresponding independent transgenic lines was used as templates. All the products were purified and mixed with equal nanomole as one sample for DNA library construction with Illumina Truseq DNA sample preparation kit (Illumina, San Diego, CA) according to manufacturer's instruction. The DNA library was not fragmented, and it was applied to the Illumina HiSeq 3000 system (paired‐end 150 bp reads, Illumina).

Off‐target sites of *GhCLA1* were analysed with a localized *G. huristum* genome database, and a Perl script ‘ot2gtf_v2.pl’ that was adapted from Xie *et al*. (2014). For sgRNA7, sgRNA8, sgRNA9 and sgRNA10, highly potential off‐target sites of 6, 4, 7 and 9 were chosen, respectively. A 300 bp DNA sequence that covered each off‐target site was used for primer design. Primers are listed in Table S4. Mixed genomic DNA from albino plants was used as the template. All the products were purified and mixed with equal amounts (50 ng for each) as one sample. DNA library construction and sequencing were conducted as the barcode‐based sequencing process.

### Data analysis

Raw data of high‐throughput sequencing was adapter‐clipped using Trimmomatic with default parameters. Replicated reads were omitted to obtain unique reads. Sequence reads for *GhCLA1* mutation genotyping were sorted with the barcode‐marked primer pairs and were traced back to original plants. Sequence reads for off‐target check were sorted with the specific primer pairs. The independent reads of each plant or each off‐target sites were aligned with software BioEdit (Version 7.0.9.0, Hall, 1999). Genome sequence of *GhCLA1* and sequences that covered each off‐target sites were used as reference, respectively.

## Supporting information


**Figure S1** Cloning of *AtU6‐26* homologous genes and their promoters in cotton.
**Figure S2** Modification of pGERB32 vector for cotton gene transformation.
**Figure S3** Procedure for pPTG vectors construction with one step cloning method.
**Figure S4** The *DsRed2* transgenic cotton line RED has red fluorescence in all tissues.
**Figure S5** Designed *PTG* genes for *DsRed2* and *GhCLA1* editing.
**Figure S6** Transgenic positivity check in the regenerated T0 plants with Cas9 specific primers.
**Figure S7** Southern blotting analysis of *DsRed2* gene copies in T0 plants and their T1 progenies.
**Figure S8** An example of off‐target effect analysis at one potential off‐target site 15_POT3782.
**Table S1** Barcoded‐primers for *GhCLA1* mutation genotyping in independent T0 transgenic lines with high‐throughput sequencing method.
**Table S2** Statistics of the sequencing reads generated from barcode‐based sequencing.
**Table S3** Statistics of sequencing reads generated for off‐target analysis.
**Table S4** Primers used for vectors construction, genotyping at on‐targets and off‐target sites.
**Appendix S1** DNA sequence of promoter *pGhU6.9*.
**Appendix S2** Genome DNA Sequences of each potential off‐target site.
