# Supplementary material for: High efficient multisites genome editing in allotetraploid cotton (Gossypium hirsutum) using CRISPR/Cas9 system
Source: Plant Biotechnol J. 2017 Jun 20;16(1):137–50. doi: 10.1111/pbi.12755 (PMC5785356; doi:10.1111/pbi.12755)
Supplement: Supplementary file 1 — Figure S1 Cloning of AtU6‐26 homologous genes and their promoters in cotton. Figure S2 Modification of pGERB32 vector for cotton gene transformation. Figure S3 Procedure for pPTG vectors construction with one step cloning method. Figure S4 The DsRed2 transgenic cotton line RED has red fluorescence in all tissues. Figure S5 Designed PTG genes for DsRed2 and GhCLA1 editing. Figure S6 Transgenic positivity check in the regenerated T0 plants with Cas9 specific primers. Figure S7 Southern blotting analysis of DsRed2 gene copies in T0 plants and their T1 progenies. Figure S8 An example of off‐target effect analysis at one potential off‐target site 15_POT3782. Table S1 Barcoded‐primers for GhCLA1 mutation genotyping in independent T0 transgenic lines with high‐throughput sequencing method. Table S2 Statistics of the sequencing reads generated from barcode‐based sequencing. Table S3 Statistics of sequencing reads generated for off‐target analysis. Table S4 Primers used for vectors construction, genotyping at on‐targets and off‐target sites. Appendix S1 DNA sequence of promoter pGhU6.9. Appendix S2 Genome DNA Sequences of each potential off‐target site. [file PBI-16-137-s001.pdf]

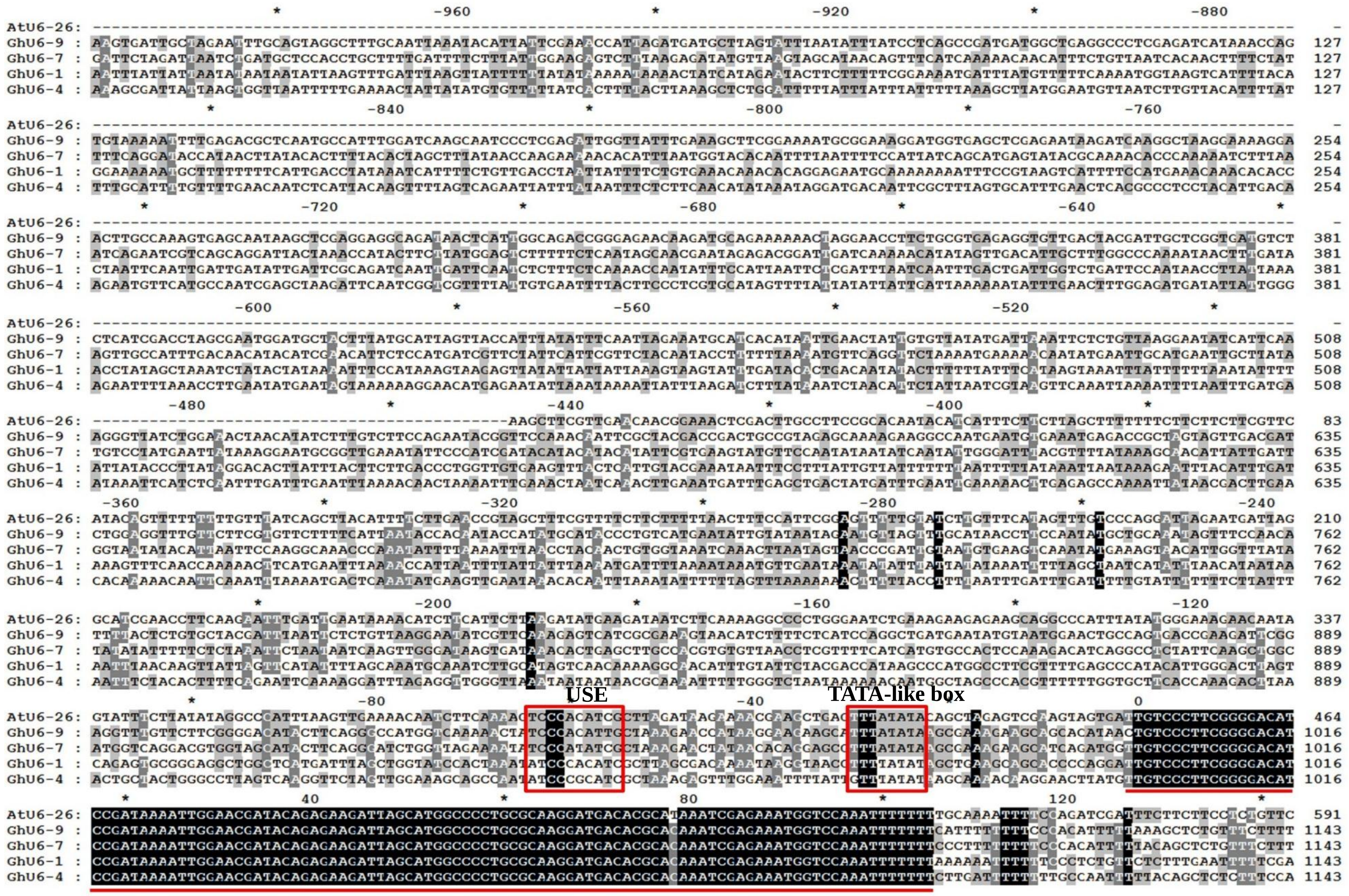

Figure S1. Cloning of *AtU6-26* homologous genes and their promoters in cotton. The conserved coding regions are in black background and underlined with a red line. Two conserved elements in the promoter region are indicated in red boxes with annotation.

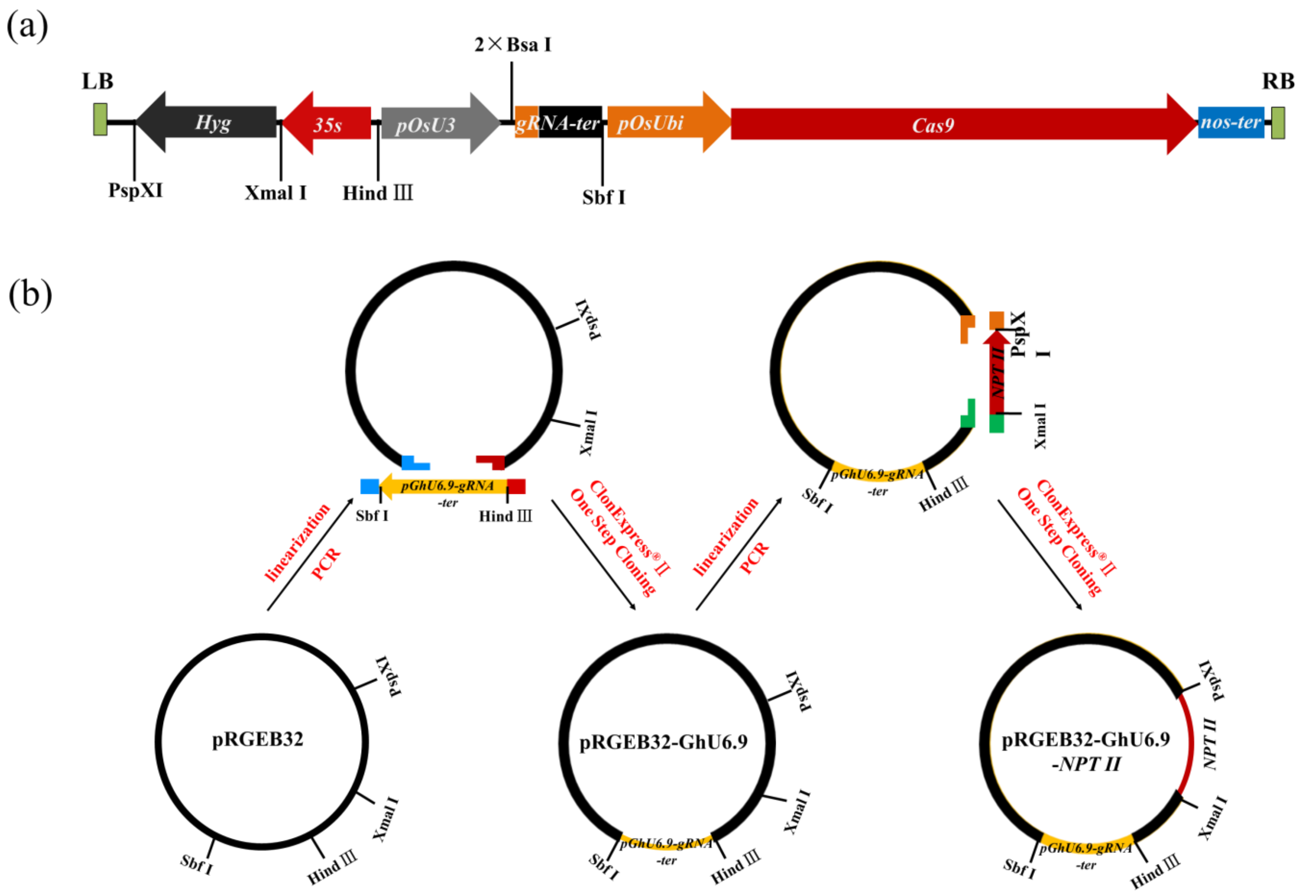

Figure S2. Modification of pRGEB32 vector for cotton gene transformation. (a) Schematic of the T-DNA region of vector pRGEB32. Restriction enzyme sites near the selection marker *Hyg* and *pOsU3* are labeled below. Two reverse Bsa I sites are the cloning sites to insert sgRNAs. (b) Detailed procedure of vector modification is illustrated. PCR adaptors that are homologous to the linearized vector are shown in same color.

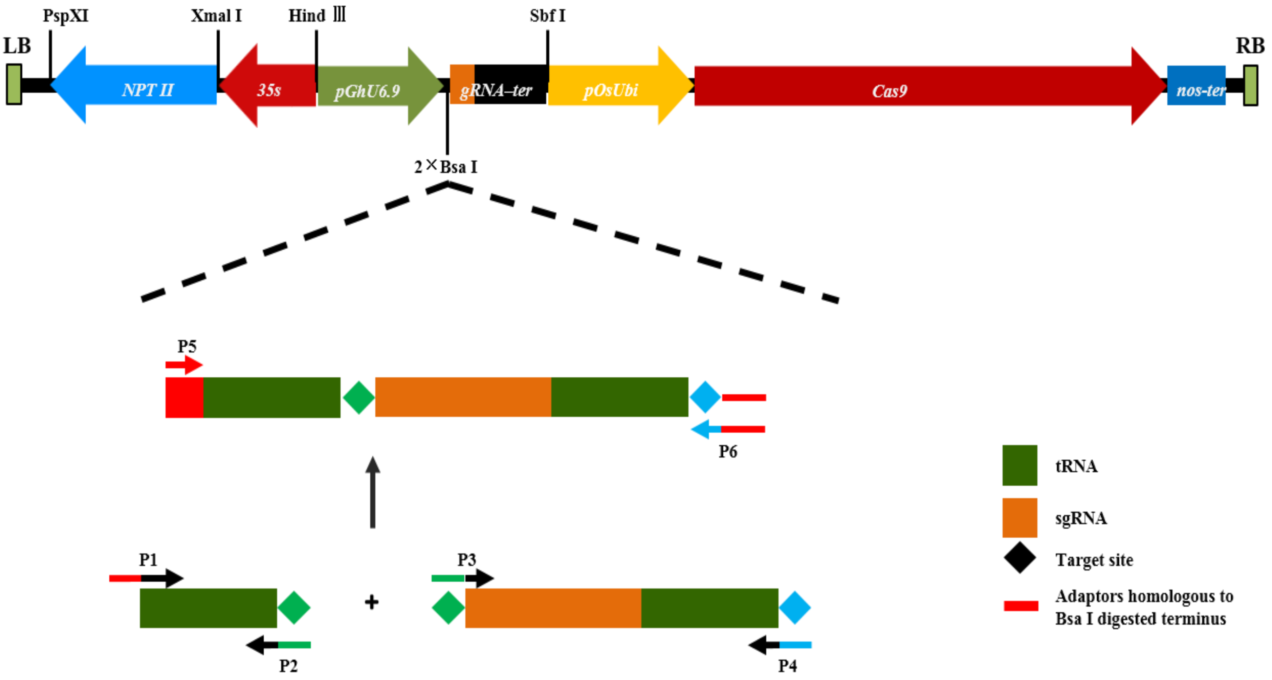

Figure S3. The procedure for pPTG vectors construction with one step cloning method. Above diagram shows the T-DNA region of expression vector pRGEB32-GhU6.9-NPT II. To combine two sgRNAs in one vector, two separate PCR products are obtained with primer pairs P1-P2 and P3-P4, respectively. These two fragments are assembled with overlapping extension PCR with primer pair P5-P6. The final PCR product is designed to be inserted into the Bsa I linearized expression vector using one step cloning method.

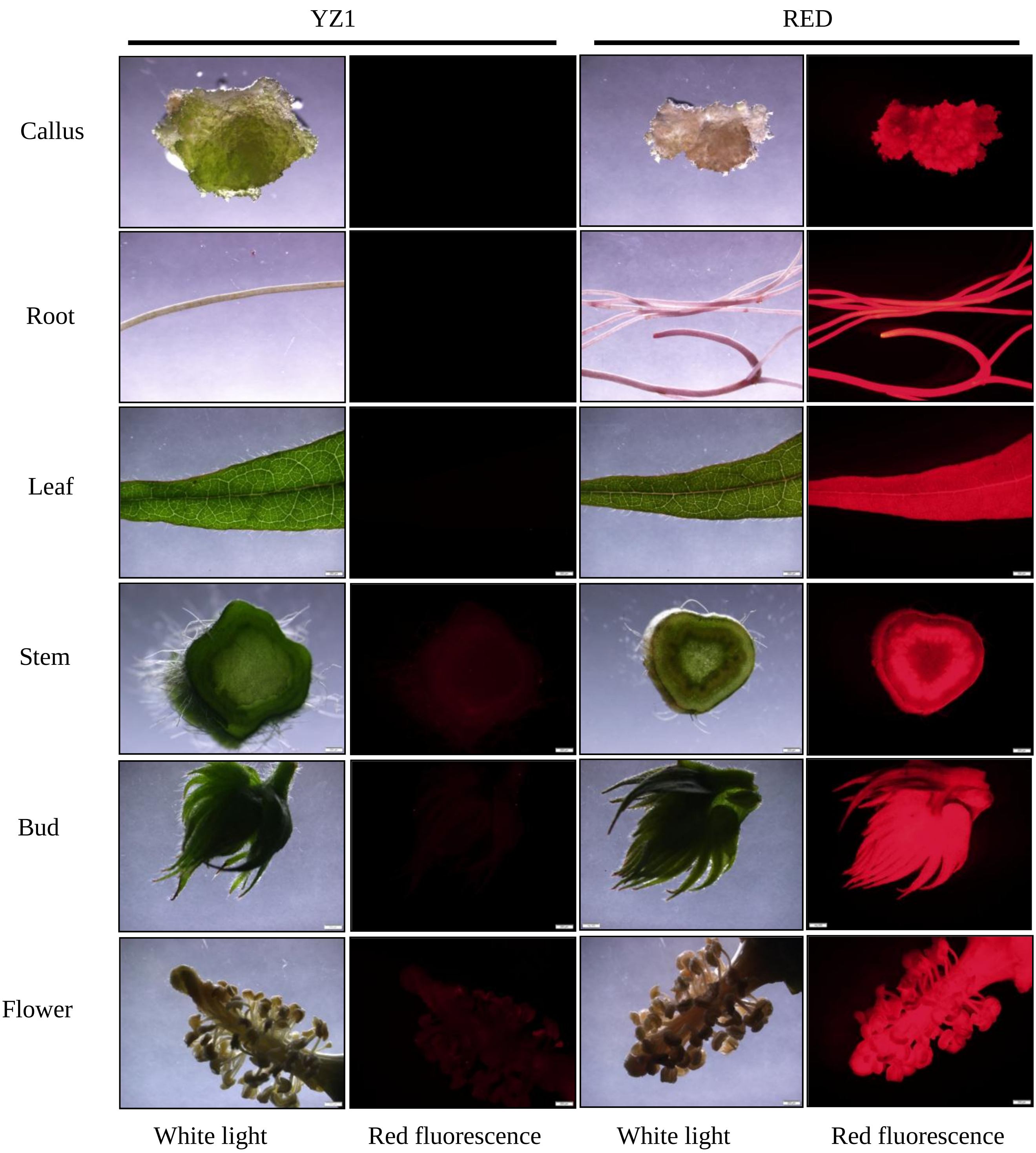

Figure S4. The *DsRed2* transgenic cotton line RED has red fluorescence in all tissues. Samples were observed under a stereomicroscope in the bright field and the red fluorescence field at an excitation wavelength of 530nm to 550nm.

(a)

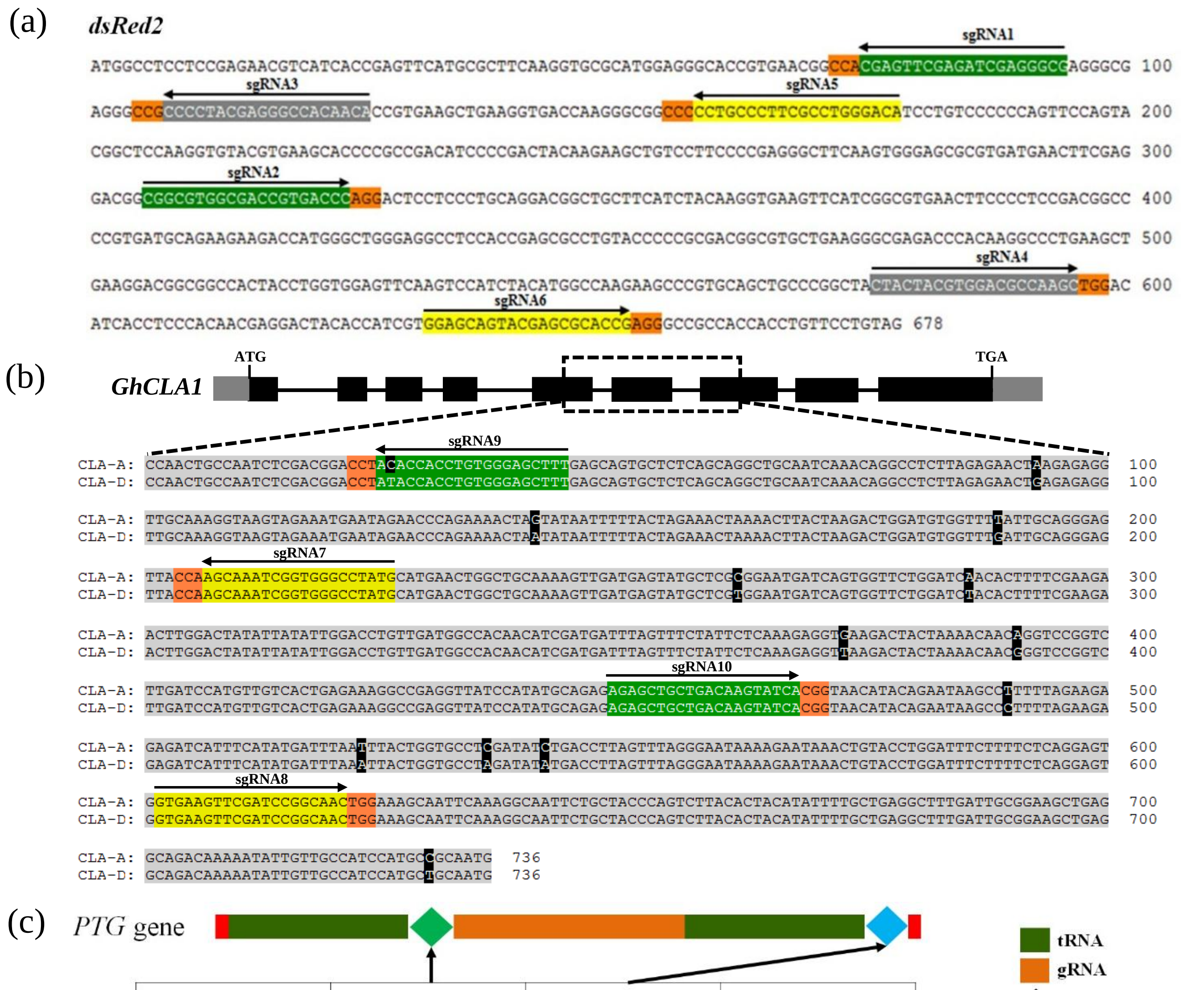

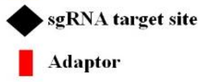

PTG genes	Target site1	Target site 2	Target gene
PTG1	sgRNA1	sgRNA2	*dsRed2*
PTG2	sgRNA3	sgRNA4	
PTG3	sgRNA5	sgRNA6	
PTG4	sgRNA7	sgRNA8	*GhCLA1*
PTG5	sgRNA9	sgRNA10	

Figure S5. Designed *PTG* genes for *DsRed2* and *GhCLA1* editing. (a) The designed sgRNA target sites to *DsRed2*. (b) The designed sgRNA target sites to *GhCLA1*. The sgRNA target sites are highlighted in yellow or green, with the recognition direction indicated with arrows. sgRNAs chosen to be combined in one vector are illustrated in the same color. The PAM regions are highlighted in orange. Different SNPs in *GhCLA1* DNA sequences are highlighted in black. (c) The illustration of a *PTG* gene. Compositions of different *PTG* gene for the target genes are listed.

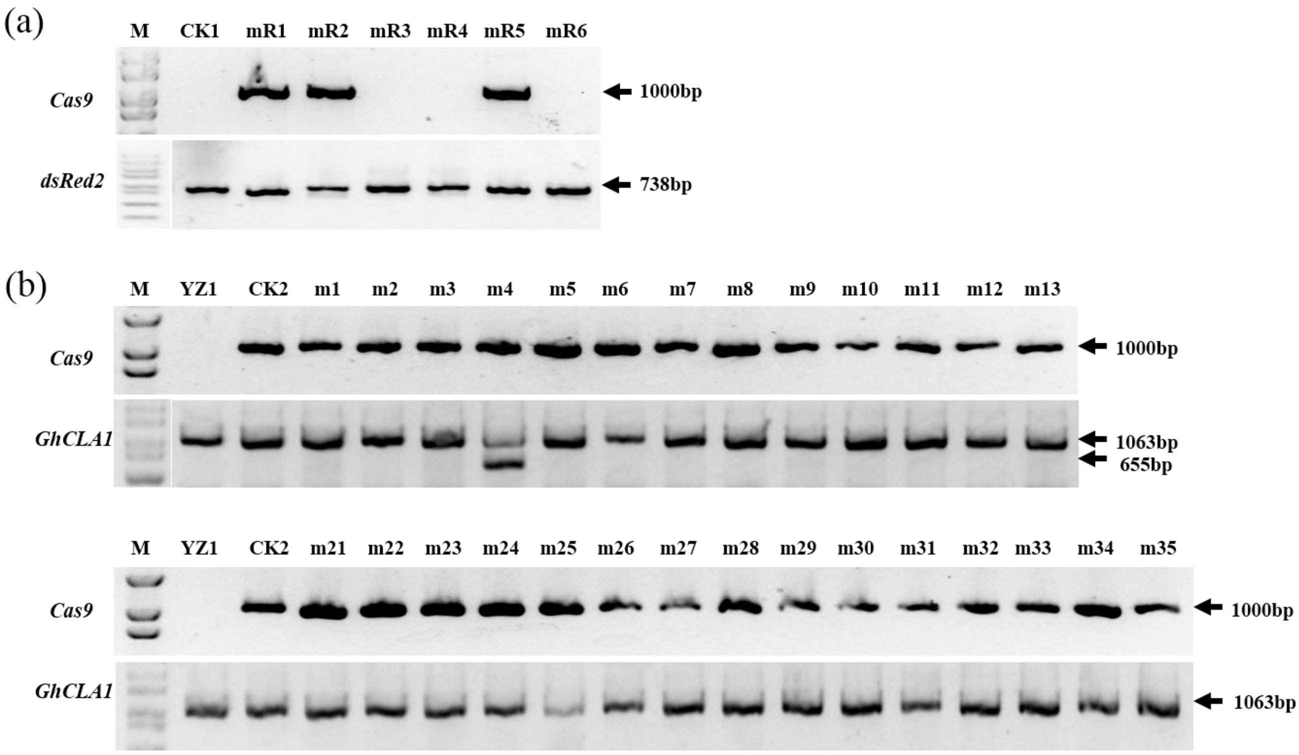

Figure S6. Transgenic positivity check in the regenerated T0 plants with Cas9 specific primers. (a) PCR analysis of *Cas9* positivity and *DsRed2*. (b) PCR analysis of *Cas9* positivity and *GhCLA1*. M, marker. CK1, a Cas9 positive control without sgRNA targeting in *DsRed2* transgenic background. CK2, a Cas9 positive control without sgRNA targeting in YZ1 background.

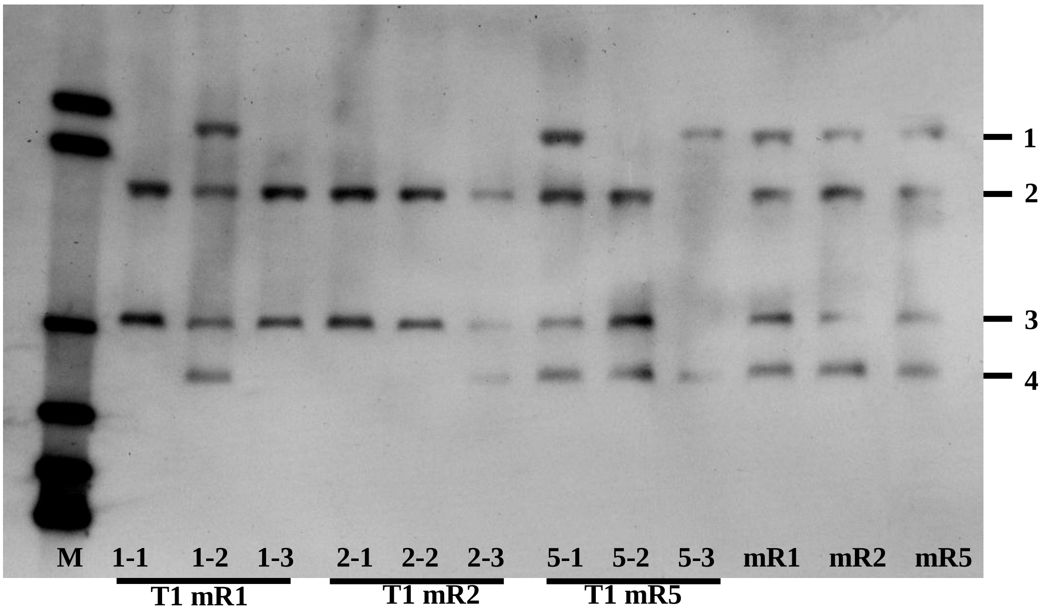

T0 Lines	*DsRed2* copies	Mutation type at sgRNA5	T1 progenies	*DsRed2* copies	Mutation type at sgRNA5
mR1	1, 2, 3, 4	+G , -G	1-1	2, 3	+G
			1-2	1, 2, 3, 4	+G , -G
			1-3	2, 3	+G
mR2	1, 2, 3, 4	+G , -G	2-1	2, 3	+G
			2-2	2, 3	+G
			3-3	2, 3, 4	+G , -G
mR5	1, 2, 3, 4	+G , -G	5-1	1, 2, 3, 4	+G , -G
			5-2	2, 3, 4	+G , -G
			5-3	1, 4	+G , -G

Figure S7. Southern blotting analysis of *DsRed2* gene copies in T0 plants and their T1 progenies. M, marker. mR1, mR2 and mR5 represents the three T0 cotton lines. Three independent T1 progenies for each T0 lines were analyzed. Number 1 to 4 represents the independent gene copies of *DsRed2*. A summary of the Southern result and mutation genotype of T0 and T1 plants is listed. The T1 progenies inherited *DsRed2* mutations from corresponding T0 plants. *DsRed2* copies of 1, 2 and 3 have +G mutation type. The copy 4 carries -G mutation type.

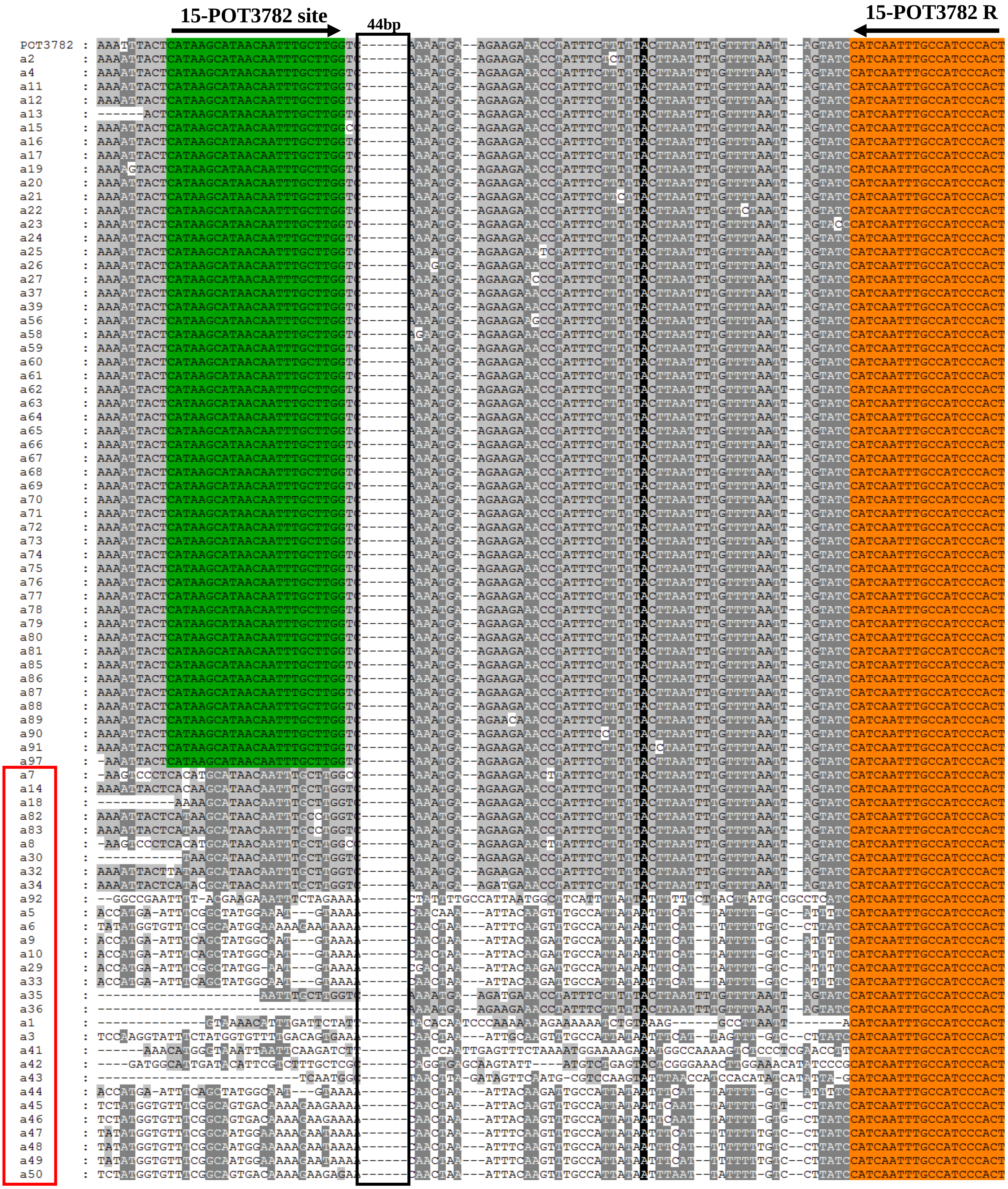

Figure S8 An example of off-target effect analysis at one potential off-target site 15_POT3782. The potential off-target site and the reverse primer for PCR process are highlighted in green and orange background, respectively. The gap of omitted nucleotides is labeled above. The reads in red box were unpredicted sequences that might be generated because of mismatch of primers.

Table S1. Barcoded-primers for *GhCLA1* mutation genotyping in independent T0 transgenic lines with high-throughput sequencing method

	Lines	Forward primer	Reverse primer
PTG3	**m1**	**cgactg**TAAGACTGGATGTGGTGTTATTGC	**gctagt**AAGATGTAGTGTAAGACTGGGTAGC
	**m2**	**cgactg**TAAGACTGGATGTGGTGTTATTGC	**atgcta**AAGATGTAGTGTAAGACTGGGTAGC
	**m3**	**cgactg**TAAGACTGGATGTGGTGTTATTGC	**ctgcga**AAGATGTAGTGTAAGACTGGGTAGC
	**m4**	**cgactg**TAAGACTGGATGTGGTGTTATTGC	**gtactc**AAGATGTAGTGTAAGACTGGGTAGC
	**m5**	**cgactg**TAAGACTGGATGTGGTGTTATTGC	**tctagc**AAGATGTAGTGTAAGACTGGGTAGC
	**m6**	**cgactg**TAAGACTGGATGTGGTGTTATTGC	**gagtca**AAGATGTAGTGTAAGACTGGGTAGC
	**m7**	**tgatag**TAAGACTGGATGTGGTGTTATTGC	**gctagt**AAGATGTAGTGTAAGACTGGGTAGC
	**m8**	**tgatag**TAAGACTGGATGTGGTGTTATTGC	**atgcta**AAGATGTAGTGTAAGACTGGGTAGC
	**m9**	**tgatag**TAAGACTGGATGTGGTGTTATTGC	**ctgcga**AAGATGTAGTGTAAGACTGGGTAGC
	**m10**	**tgatag**TAAGACTGGATGTGGTGTTATTGC	**gtactc**AAGATGTAGTGTAAGACTGGGTAGC
	**m11**	**tgatag**TAAGACTGGATGTGGTGTTATTGC	**tctagc**AAGATGTAGTGTAAGACTGGGTAGC
	**m12**	**tgatag**TAAGACTGGATGTGGTGTTATTGC	**gagtca**AAGATGTAGTGTAAGACTGGGTAGC
	**m13**	**gtcacg**TAAGACTGGATGTGGTGTTATTGC	**gtactc**AAGATGTAGTGTAAGACTGGGTAGC
	**yz1-1**	**gtcacg**TAAGACTGGATGTGGTGTTATTGC	**tctagc**AAGATGTAGTGTAAGACTGGGTAGC
PTG4	**m21**	**gtcacg**TCTCTGCCAACTGCCAATCTCG	**gagtca**TCTCTTCTAAAAAGGCTTATTCTGT
	**m22**	**atgatg**TCTCTGCCAACTGCCAATCTCG	**gctagt**TCTCTTCTAAAAAGGCTTATTCTGT
	**m23**	**atgatg**TCTCTGCCAACTGCCAATCTCG	**atgcta**TCTCTTCTAAAAAGGCTTATTCTGT
	**m24**	**atgatg**TCTCTGCCAACTGCCAATCTCG	**ctgcga**TCTCTTCTAAAAAGGCTTATTCTGT
	**m25**	**atgatg**TCTCTGCCAACTGCCAATCTCG	**gtactc**TCTCTTCTAAAAAGGCTTATTCTGT
	**m26**	**atgatg**TCTCTGCCAACTGCCAATCTCG	**tctagc**TCTCTTCTAAAAAGGCTTATTCTGT
	**m27**	**atgatg**TCTCTGCCAACTGCCAATCTCG	**gagtca**TCTCTTCTAAAAAGGCTTATTCTGT
	**m28**	**cagtca**TCTCTGCCAACTGCCAATCTCG	**gctagt**TCTCTTCTAAAAAGGCTTATTCTGT
	**m29**	**cagtca**TCTCTGCCAACTGCCAATCTCG	**atgcta**TCTCTTCTAAAAAGGCTTATTCTGT
	**m30**	**acgtca**TCTCTGCCAACTGCCAATCTCG	**ctgcga**TCTCTTCTAAAAAGGCTTATTCTGT
	**m31**	**acgtca**TCTCTGCCAACTGCCAATCTCG	**gtactc**TCTCTTCTAAAAAGGCTTATTCTGT
	**m32**	**acgtca**TCTCTGCCAACTGCCAATCTCG	**tctagc**TCTCTTCTAAAAAGGCTTATTCTGT
	**m33**	**acgtca**TCTCTGCCAACTGCCAATCTCG	**gagtca**TCTCTTCTAAAAAGGCTTATTCTGT
	**m34**	**gtcacg**TCTCTGCCAACTGCCAATCTCG	**tgcgac**TCTCTTCTAAAAAGGCTTATTCTGT
	**m35**	**gtcacg**TCTCTGCCAACTGCCAATCTCG	**gtcacg**TCTCTTCTAAAAAGGCTTATTCTGT
	**yz1-2**	**acgtca**TCTCTGCCAACTGCCAATCTCG	**acgtca**TCTCTTCTAAAAAGGCTTATTCTGT

Note: The 6-nucleotide barcodes were in colors and they were chosen as specific combinations to mark independent plantlets.

Table S2. Statistics of the sequencing reads generated from barcode-based sequencing

Lines	Barcode-sorted unique reads	Lines	Barcode-sorted unique reads	Lines	Barcode-sorted unique reads
m1	162	m11	181	m27	675
m2	622	m12	645	m28	61
m3	891	m13	552	m29	179
m4	817	yz1-1	329	m30	316
m5	497	m21	320	m31	257
m6	269	m22	164	m32	175
m7	56	m23	571	m33	262
m8	192	m24	724	m34	181
m9	329	m25	556	m35	93
m10	257	m26	434	yz1-2	78
**Total barcode-sorted unique reads**				10845	
**Unique reads**				137545	
**Unknown reads**				99054	
**Total reads**				200687	

Table S3. Statistics of sequencing reads generated for off-target analysis

Off-target site	Primers-sorted unique reads	Off-target site	Primers-sorted unique reads
15_POT1500	258	21_POT7274	414
15_POT2941	340	21_POT7350	224
15_POT3515	415	21_POT9661	244
15_POT3782	108	21_POT10018	447
15_POT9381	185	28_POT25	370
15_POT12615	171	28_POT26	124
36_POT585	385	28_POT198	116
36_POT4307	122	28_POT456	189
36_POT8435	141	28_POT1542	235
36_POT11074	178	28_POT4528	123
21_POT1840	289	28_POT4625	276
21_POT3811	244	28_POT6578	569
21_POT6973	444	28_POT7818	361
**Total primers-sorted unique reads**		6972	
**Unique reads**		337594	
**Unknown reads**		330622	
**Total reads**		953687	

Table S4 Primers used for vectors construction, genotyping at on-targets and off-target sites

**Primers**	**Sequences (5' to 3')**	**Purpose**
pGhU6.9 F	TGATTGCTAGAATTTGCAGTAGGCTT	pGhU6.9 promoter amplification
pGhU6.9 R	AGTTATGTGCTGCTTCTTTCGCTTAT	
midU6-9 R	TAGCCGTCTCATTTCACATTCATTG	pGhU6.9 mutation to destroy the Bsa I site
midU6-9 F	CAATGAATGTGAAATGAGACGGCTA	
infU6-9 F	TGATTACGCCAAGCTTTGATTGCTAGAATTTGCAGTAGGCTT	Overlap-extension amplification of pGhU6.9-gRNA-ter for ligation to pRGEB32
infU6-9 R	AAACCGAGACCTCGGTCTCCTGCCAGTTATGTGCTGCTTCTTTCGCTTAT	
gRNA-ter F	GGCAGGAGACCGAGGTCTCGGTTT	
gRNA-ter R	CCGAATTTGTGGACCTGCAGGCATG	
inf32B-Kan F	TTTCGCAGATCCCGGGATGATTGAACAAGATGGATTG	*NPT II*change at the *Hpt* site
inf32B-Kan R	TATGGAGAAACTCGAGCTCAGAAGAACTCGTCAAGAAG	
PTG-RED1-1 R	CCTGCCCTTCGCCTGGGACAtgcaccagccgggaat	*PTG1* construction for *DsRed2* targeting
PTG-RED1-1 F	TGTCCCAGGCGAAGGGCAGG**gttttagagctagaaata**	
PTG-RED1-2 R	CGGTGCGCTCGTACTGCTCCtgcaccagccgggaat	
PTG-RED1-2 F	GGAGCAGTACGAGCGCACCG**gttttagagctagaaata**	
infRED1 R	**ttctagctctaaaac**CGGTGCGCTCGTACTGCTCC	
PTG-RED2-1 R	CGAGTTCGAGATCGAGGGCGtgcaccagccgggaat	*PTG2* construction for *DsRed2* targeting
PTG-RED2-1 F	CGCCCTCGATCTCGAACTCG**gttttagagctagaaata**	
PTG-RED2-2 R	GGGTCACGGTCGCCACGCCGtgcaccagccgggaat	
PTG-RED2-2 F	CGGCGTGGCGACCGTGACCC**gttttagagctagaaata**	
infRED2 R	**ttctagctctaaaac**GGGTCACGGTCGCCACGCCG	
PTG-RED3-1 R	CCCCTACGAGGGCCACAACAtgcaccagccgggaat	*PTG3* construction for *DsRed2* targeting
PTG-RED3-1 F	TGTTGTGGCCCTCGTAGGGG**gttttagagctagaaata**	
PTG-RED3-2 R	GCTTGGCGTCCACGTAGTAGtgcaccagccgggaat	
PTG-RED3-2 F	CTACTACGTGGACGCCAAGC**gttttagagctagaaata**	
infRED3 R	**ttctagctctaaaac**GCTTGGCGTCCACGTAGTAG	
PTG-CLA1-1 R	AGCAAATCGGTGGGCCTATGtgcaccagccgggaat	*PTG4* construction for *GhCLA1* targeting
PTG-CLA1-1 F	CATAGGCCCACCGATTTGCT**gttttagagctagaaata**	
PTG-CLA1-2 R	GTTGCCGGATCGAACTTCACtgcaccagccgggaat	
nPTG-CLA1-2 F	GTGAAGTTCGATCCGGCAAC**gttttagagctagaaata**	
infCLA1 R	**ttctagctctaaaac**GTTGCCGGATCGAACTTCAC	
PTG-CLA2-1 R	ATACCACCTGTGGGAGCTTTtgcaccagccgggaat	*PTG5* construction for *GhCLA1* targeting
PTG-CLA2-1 F	AAAGCTCCCACAGGTGGTAT**gttttagagctagaaata**	
PTG-CLA2-2 R	TGATACTTGTCAGCAGCTCTtgcaccagccgggaat	
PTG-CLA2-2 F	AGAGCTGCTGACAAGTATCA**gttttagagctagaaata**	
infCLA2 R	**ttctagctctaaaac**TGATACTTGTCAGCAGCTCT	

Table S4 continued		
pG32-9 F	CAGCACATAACTGGCAaacaaagcaccagtggtctag	Infusion ligation of *PTG* genes to expression vector
infpG32-9 F	CAGCACATAACTGGCAAACAAA	
u6-9 F	GTCAAAAACTATCCCACATTGCTAA	Clone verification with PCR and sequencing
ubi2 R	CTGGCTTCCGCCTTGACCCG	
gtCas9 F	GCTTGTGCGTTTCGATTTGA	Transgenic *Cas9* positivity verification
gtCas9 R	CCGCTCGTGCTTCTTATCCT	
35S F	GTGGAAAAGGAAGGTGGCT	*DsRed2* positivity check
RED R	CTACAGGAACAGGTGGTGGCGGCCC	
CLA1 F	GGATGGCTGTGGGAAGGGATCTGAAAGGTG	Full length *GhCLA1*, TA cloning for sgRNA9-sgRNA10 editing check
CLA1 R	CATAAGCCCTTTGCATGAATGATGAGTAGAT	
sCLA-1 F	CTCGACGGACCTATACCACCTGTGG	TA cloning for sgRNA7-sgRNA8 editing check with primer 'CLA1 R'
sgR9 R	GCCTTTCTCAGTGACAACATGGATC	T7E1 assay for target sgRNA9 with primer 'CLA1 F'
sgR7 F	GGTAAGTAGAAATGAATAGAACCCAGAAAA	T7E1 assay for target sgRNA7 with primer 'sgR9 R'
sgR8 F	TTTAGTTTCTATTCTCAAAGAGGTTAAGACT	T7E1 assay for target sgRNA8
sgR8 R	GTTTAATCCGGTTCCACCTCCCATTGCAGCA	
sgR10 F	TTTAGTTTCTATTCTCAAAGAGGTTAAGACT	T7E1 assay for target sgRNA10
sgR10 R	GACTGGGTAGCAGAATTGCCTTTGA	
28_POT25 F	GACTTCGCTTCATTGCCTGA	Off-target site amplification
28_POT25 R	TCTTATGGTCGAGTCTGACGAA	
28_POT26 F	TGCATGTGGTAGAGCTTCTGA	
28_POT26 R	TTTTACACAGATTCAATCAAACACC	
28_POT198 F	GGGTCGCCAGTTAGAAGAAA	
28_POT198 R	TTGTGAACTTGGTTGGCAAG	
28_POT456 F	TTTGTTTTATTGCCCGAGGA	
28_POT456 R	ATCCGGATGTTATGGTCGAG	
28_POT1542 F	GCCTGAGGATTCAAGCTAACA	
28_POT1542 R	CGATCTTTTTCTTCATATCTGGTTG	
28_POT4528 F	GCATAACTGGCAGCAAGGAT	
28_POT4528 R	TGGTCACACCCTGGAGCTAT	
28_POT4625 F	TGGCTTTTCTACACTGCTCAA	
28_POT4625 R	CGGATGTGCCAGGATTAGAT	
28_POT6578 F	TTCAATCATCTCGCCAAACA	
28_POT6578 R	AGCAATATGCCCAAAGATGG	
28_POT7818 F	TGCAAGAGCTGTCAAGGATG	
28_POT7818 R	TCAAAAGCAACAACGTTTCA	
36_POT585 F	CAACTACTGCACAGGCCAAA	
36_POT585 R	AAAATAAAATTCTCGTCTGACATGAT	

36_POT585 R	AAAATAAAATTCTCGTCTGACATGAT	**Table S4 continued**
36_POT4307 F	TGGTTGCAAACAAAGAGGACT	
36_POT4307 R	CCTTTGGCTGCTCTGTTGAC	
36_POT8435 F	GTTTCAAATAAAAAGGAGGTTTGC	
36_POT8435 R	ACAAATTTAACTCTTAACCTTTACACG	
36_POT11074 F	TCACCACATGAACCAAAATCA	
36_POT11074 R	CTTACTCTGTTTCTTTTCCCATGTT	
21_POT1840 F	TTGTGCTGCTGTTTCTTTGC	
21_POT1840 R	CCAAATGGCCCAAAAACTAA	
21_POT3811 F	TTTTGTCTTGCAACACTTTTGG	
21_POT3811 R	CTGAGCTTCCCAGTTTGTTTG	
21_POT6973 F	TCAAACGAGGATCTTTTCACG	
21_POT6973 R	TGGCCTCTTTATGTTTCTAGCAT	
21_POT7274 F	GCATTACAATGCTCCCTGGT	
21_POT7274 R	CAACAACAAGTGAGTTTATCACACA	
21_POT7350 F	CAAGCATCAGGTAGGGCATT	
21_POT7350 R	CCTACCTTGACACGCCTTCT	
21_POT9661 F	TTCATGAACAACAGGAATGATTG	
21_POT9661 R	CCGACTGTTTTGAACCCATT	
21_POT9729 F	AGAGGCCTGTTTGATTGCAG	
21_POT9729 R	TGGTGCTATGACTGCAGGAC	
21_POT10018 F	TTCACAAGGGATTTTAGTTGTTCA	
21_POT10018 R	CAACAAGTTTCTTCAAGGCTCA	
15_POT1500 F	CACGAGTTTCCCAAACTTCA	
15_POT1500 R	CAGACAGGATCCCAATTTCAA	
15_POT2941 F	CATGATTAATTGTCATGAACTGCT	
15_POT2941 R	TGAAAATTGGGCTGTTACAAAA	
15_POT3515 F	ACGGCCGTGACATTTTATTG	
15_POT3515 R	AGGCAACCCGAGCACTAACT	
15_POT3782 F	TCAATGGCAACTTCCAAAACT	
15_POT3782 R	AGTGGGATGGCAAATTGATG	
15_POT9381 F	AACAACTAATTGGTCCAAAATCA	
15_POT9381 R	GGGTAAATGGATCCCTCCTC	
15_POT12615 F	CTAGGGGCTAGCCGTTTGAT	
15_POT12615 R	TCGCAGAGCACTTCAATAGC	

Notes: The nucleotide in red capital is a mutation to destroy Bsa I site of original pGhU6.9. The nucleotides highlighted in yellow background represent its overlap with terminus of the digested expression vector. Nucleotides in blue or red lower case represent its match with gRNA and tRNA, respectively.

Appendix S1 DNA sequence of promoter *pGhU6.9*

>pGhU6.9

TGATTGCTAGAATTTGCAGTAGGCTTTGCAATTAAATACATTATTCGAAACCATTAGATGA
TGCTTAGTATTTAATATTTATCCTCAGCCGATGATGGCTGAGGCCCTCGAGATCATAAACC
AGTGTAAAAATTTTGAGACGCTCAATGCCATTTGGATCAAGCAATCCCTCGAGATTGGTT
ATTTGAAAGCTTCGGAAAATGCGGAAAGGATGGTGAGCTCGAGAATAAGATCAAGGCTA
AGGAAAAGGAACTTGCCAAAGTGAGCAATAAGCTCGAGGAGGCAGATAACTCATTGGC
AGACCGGGAGAACAAGATGCAGAAAAAACTAGGAACCTTCTGCGTGAGAGGTGTTGAC
TACGATTGCTCGGTGATGTCTCTCATCGACCTAGCGAATGGATGCTACTTTATGCATTAGT
TACCATTTATATTTCAATTAGAAATGCATCACATAATTGAACTATTGTGTTATATGATTAAAT
TCTCTGTTAAGGAATATCATTCAAAGGGTTATCTGGAAACTAACATATCTTTGTCTTCCAG
AATACGGTTCCAAACAATTCGCTACGACCGACTGCCGTAGAGCAAAAGAAGGCCAATGA
ATGTGAAATGAGACCGCTAGTAGTTGACGATCTGGAGGTTTGTTCTTCGTGTTCTTTTCA
TTAATACCACAATACCATATGCATACCCTGTCATGAATATTGTATAATAGAATGTTAGTTTG
CATAACCTTCCAATATGCTGCAAATAGTTTCCAACATTTTACTCTGTGCTACGATTTAATTC
TCTGTTAAGGAATATCGTTCAAAGAGTCATCGCGAAAGTAACATCTTTTCTCATCCAGGC
TGATGAATATGTAATGGAACTGCCAGTGACCGAAGATTCGGAGGTTTGTTCTTCGGGGAC
ATACTTCAGGGCCATGGTCAAAAACTATCCCACATTGCTAAAGAACCATAAGGAAGAAG
CATTTATATAAGCGAAAGAAGCAGCACATAACT

Note: The highlighted "GAGACC", which was a Bsa I recognition site, is mutated to "GAGAC**G**" to destroy the recognition site to avoid more than two specific Bsa I sites in the final expression vector.

Appendix S2 Genome DNA Sequences of each potential off-target site

>CLA_A_15_POT1500

ATTTTTCAATAACCGGAGACAAGGATAACACGAGTTTCCCAAACTTCATAGCCTCTCAACAAATCTTA
AAAATTTCATGCAAAATAATATATATATATATAAAAGGACCATTGATTTGCTTGGATGGAGACACCCCTAT
GCTGATGTTGGAGTCTCTTAAATCTCCACTTGCTTCTATGTATAAATTGACTTGTGTTGAGCATCAAATA
TCTTGAAATTGGGATCCTGTCTGTATTTATATTCTTTGGACT

>CLA_A_15_POT2941

TACCTATCAAAATATGTACAATTAATATAAAACATTAATGTCATGATTAATTGTCATGAACTGCTAACTAT
CAAAATTCATAAGATCTCCAATTTGCTTGGAAAGGTCCTTTTACACCAAAAAAAAGGTTAAAATATGTT
ATAAGTCCTTGTAGTCTTTGTAAATTTAAAGTTTAGCCTCTATACTTTTGTAACAGCCCAATTTTCAGTA
GTGTCAGAACAGTGATTTGAGATCACTAAATTCGATGAAGA

>CLA_A_15_POT3515

GACCGTGGCCATGTCGCACGGCCGTGACATTTTATTGTAGCTCGAGTTTGGGAAAATAGTTTCCCTATT
TTCACGTGGCCCTAAGCACGCCCATTTGCTTGGCCGTGTCTCTGTGGAAAACCTGTATTCAAGAGCTC
CATTAGTAAGTTAGGTGTTGAAGACTAAATTTTAAAGAAGTTAATACAGTTAGTGCTCGGGTTGCCTGC
CGAGAAGCGCTTATTTGTAGTCTAAGCTCGACTTACCTCTCCATTG

>CLA_A_15_POT3782

TTCATAAAAAATATCTAATATCCATCAATGGCAACTTCCAAAACTTAAAAATTACAAGAAACAGGTAAA
AAAATTTACTCATAAGCATAACAATTTGCTTGGTCGACTTTCCTAGCTTGAAAAACCATGAATTTCGGC
TATGGAATGTAAAATGAAGAAGAAACCTATTTCTTTTTACTTAATTTTGTTTTAATTAGTATCCATCAATT
TGCCATCCCACTATACTATTTAGGGCCTAATTATCACTTAAGT

>CLA_A_15_POT9381

CTAATATTATAGTATGTGATATAGTAATACATAATGTAATAATATATTTTAAAACTATTAATACATATATATA
ACAACTAATTGGTCCAAAATCATAACTAAATTTACCAACCGATTTGCTTGGATTGGCTCGGTTTGGTTG
GAATCGGTTTTAATTGCTAATTATAATAGATTATATACAATTGCATAATATGTACTATTACATAATTTATTAA
AATAGTATATGAGGAGGGATCCATTTACCCCTGTGTA

>CLA_A_15_POT12615

ATTTTGCAACTGAGTTAAATGATAAATTGTTGTAAAATCTTATTCTAGGGGCTAGCCGTTTGATTGAACA
TTTGCATGCAAAAGGAGTACCGATTTGCTTGGCAACAGGGTAAGCATTGTCATTTCCAGTTCTTATTCC
CTTTAGTTGCTTTACTCAAAACTAAACATGGAGTGCATACACTAGTGTGAATGAGTTTTGGGTTCCTTA
ACCTTAATGGAATTGTTTAAGCTATTGAAGTGCTCTGCGAGCAT

>CLA_A_21_POT1840

TCGAAGCAAATACAACCATTGTACTTTTCGTTTTTGTTCTTTTGTGCTGCTGTTTCTTTGCCCTTGTCTC
GTGTCTGTGCAGGTACAGAGGCCAGATGGCGTGGAGGCTCGCAGTGGTGGTATAGGGAGTATGGCGG
TGACGTGGCACTAGAGGCTGGAGGTTGCGGCGCACCTAGGGCTAGGGTTCCCTAGGTTGGCTGAAAT
TGTTTTAGTTTTTGGGCCATTTGGGCCTTGTATTTTTAGGCTAGGGTTTACTTCATTTTGGGCCAT

>CLA_A_21_POT3811

AAAATCTAAACCATCAAAATGAGGACAAACTAACTTGTAAGTTTTGTCTTGCAACACTTTTGGTTAGG

TTACTTGCTACACTTCTGGTGGAGATACTTAGGGAAAAGCTCCCACAGGTTCTTTAAGAGGGAAACCA

GGTGGTAGGCCTCCCAGTACTCCTTTTCCTTTGTTCATAGCAGTAGCATTGTTGGAGGACACAAGGTG

CCCAAAATACTATTCAAACAAACTGGGAAGCTCAGATCTAAACTCCCCCTTAAACTCTTCCTTAAAC

>CLA_A_21_POT6973

ATCTCGCCTTATCTCACCTCTATTTCAAACGAGGATCTTTTCACGGTCATGGCATAGGTCTCAGAGATTT

CATCTATTGAGCAAAGTCTTTGGGTGGGGATTTTAGAAGTGCCAGCAGGTGGTATAGGAATAAACTATT

TGACTTAAAAAAGGCAGTGAAAGTTGGCGAAGACTCATTAGCTAAACTATGCTAGAAACATAAAGAG

GCCAATATCTTTGTCTAGAAAATGGACACATAACTCTCAATCATAAGAAGGAAACACAAAGAGGT

>CLA_A_21_POT7274

CGATTAGGATACGATTTGCATGCCAATAAGGTTAGAACGTGCGCCTGTATGGGATTTGCACTTCAGTGC

CCCTGTTAGCATTACAATGCTCCCTGGTGTGGTATAGGTAACCATGTGTTTGAGTTATGTTTGGTATAGT

TCATCGGGCTCCTAATATTCTTACTATGAAATTGTTTGAATGATGTTGAATACCGATTCTGAATTAGATAA

ATGAGAGTGGAATGACATAAATGTTATATGATTCATGTGTGATAAACTCACTTGTTGTTGT

>CLA_A_21_POT7350

GTAATGCGCCGGTCAAGCATCAGGTAGGGCATTTTCATGCACTAGTTGACGGACTCATGCAGCACCTT

CGGCACTCAAAGAAATCAAAAGCTTCCAAAGGTGGGATGGGATGGCTAGGAAAGAACCAACATAAA

GAAAACAGGAAGGTGAAGCAATTACATTGGTGGCAGATGTTTCAGCGTCAAGGTGGAGTAAAATTAC

CCAATAGAAGGCGTGTCAAGGTAGGGTTAATGAGTAAAAAAAAGCAGCTTAGGGTACCTAAGTTGAA

ATA

>CLA_A_21_POT9661

CAGTCGCATATGCCCTGGTGCCATCTTGAACTATCTGATGCTTAAGAGAAACATAAAATTCATGAACAA

CAGGAATGATTGTGTTTTCTTATGGAATGATACAAAATCGCTCCCAATGGTGGTATTGGACTAAAGTCT

ATATTTCCTTGCAGTGGGCCATACAAGGTTCAAATCCTCATTCTTGTATAATAGGTCTCCCTTATGATTTG

AGAAAATATTTTTCAACATTCAAATGATGAAATATTAATGGGTTCAAAACAGTCGGGGTTTC

>CLA_A_21_POT9729

TTCTATTCATTTCTACTTACCTTTGCAACCTCTCTTAGTTCTCTAAGAGGCCTGTTTGATTGCAGCCTGC

TGAGAGCACTGCTCAAAGCTCCCACAGGTGGTGTAGGTCCGTCGAGATTGGCAGTTGGCAGAGAAAC

TTGTTTATTGTCATTAAGAATAACAATCATATCAGAATCAAGATATCCGGCATTGTTCATTGCTTCGTAA

GCTTGTCCTGCAGTCATAGCACCATCACCTATGACAGCAACAACATGATTCCTTTCACCTTTCAG

>CLA_A_21_POT10018

TTTCACAAGGGATTTTAGTTGTTCATTTATCATGTCTCATTTATTTCACATTAGTTAATATGCATAACATTT

ATTCAATTTATATCAAAATATAAAATTCAAGAATAAAATTATCACATGTGGTATAGGAGATTATAGACTTT

TACTACTTTAGTTTCCTTGAGCCTTGAAGAAACTTGTTGATCAAATTCCTAAGAATGACATACGTAGTTT

ACAAGTCTGATGGTGATCTTCTTCATTCTTCCTTCTTGGCGCCATGTTTCCTTACAAT

>CLA_S_28_POT25

AGTGGATTATAATACGGTTGTCTAAATATGATCCAAGTGAGCACATGAAAGAAGACTGATAGCCCGTG

AAGAAATACAAGACTTCGCTTCATTGCCTGAAGACAGAAACTGACAAAGATTATTCGAGAGCCGTCA

CATCTTGACCTTCTTAAAGGAGCTGACAAGTATCACAGTAATAAGTGAGCAACGAGTCGCAGCCTGGA

TCGAGCAAAAAAAGGAGTTAATATAGACATCTCTTGGAGAATTCGTCAGACTCGACCATAAGATCATG

ATATGAAGAGGAAGATAGGTGTCTTCTCTT

>CLA_S_28_POT26

ATACTAAAATAAAAAATACATACCTCCTTAGAAGATCAATAGAATACTACAAAATTTAAAAGTAATAAA

AAATTAATAAAAAACAATATTTATATGTTTTCTTAAAAGGTCACTAACATGCATGTGGTAGAGCTTCTGA

CAAGAATAAGGGTCTGTTTGATTGCTAGTAAAATGTTTTTCGTAAAATGATTTCTGGAAAATGTTTTAC

TTTTTTGTAAAATAATTTACTGAAAAATATTTTCTGGTGTTTGATTGAATCTGTGTAAAATATTTTCTACT

GTTTGACNGTAAAATATTTTCT

>CLA_S_28_POT198

AAGGTGGGAATTGAAAGAGTGGGTACTATGGGGTTGCATTGTGGGTATTAGTTCTTCAAGGAAAATAG

GAGATTAGGAAGAGATTGTATTATGGTATGGACCCATAGTTACGACAATCAATTGAGTAAATGCGAAGG

GTCGCCAGTTAGAAGAAATGGATACAAGTATCACGGATGTGAAATATCGATAATCAGGGGTGAAACCT

ATGAAAGAACATGGTTTGGGCCAGACCAGGGTCAATGTCTGCAAATGAATGGCTCAGAGAGCATGAG

GCATTAGCCTTGCCAACCAAGTTCACAAA

>CLA_S_28_POT456

GTGGATTATAACGCGGTTGTCTAAATATGGTCCAAGTAAACACGAAGAAAGAAGGCTGATAGCCCATG

AAGAAATACATGACTTTGTTTTATTGCCCGAGGATTAAAATCAATAAAGATTATTCAAGAGCCGTCACG

TCTTGACTTTCTTAAAGGAGCTGACAAGTATCATGGGGATAAGTGAACAACGAGTCGCAGCCTGGATC

AAGCAAAAAGGAGTTCATGGGGATGTCTTTTGGAAAATTCACAAGACTCGACCATAACATCCGGATAT

GAAGAGGAAGATAGGTGTCTTCACTTGA

>CLA_S_28_POT1542

CTTGATAAAGTGGATTATAACACGGTTGTCTAAATATGTTCCAAGTAAACACAATGAAGGAAGATTGAT

ATTCCATGAAGAAATACAGGACTTTGCTTTATAGCCTGAGGATTCAAGCTAACAAAGATTATTCGAGAG

CTGTCATGTCTTAAAGAAGCTAACGAGTATCACGGGGATAAGTGGGCAATGGGCCGCGATCCAGATCG

AGCAGAAAGAAAATAATAGAGATATCTTTTGGAAAATTCGTAAGATTTGACCATAACAACCAGATATG

AAGAAAAAGATCGATATCTTCGCTTGG

>CLA_S_28_POT4528

ATATGTTAGGATGCACATTGTAAAGGCTACAATAGAAGACAAACAACAAAAGATCTGCCAAGATCAAA

GGCATAACTGGCAGCAAGGATCAAGGGATTAAGAAGGCAAGATATGCAAAAATTAAGGGCAGAACTG

GAAACAAGTATCAAGGGATTAAAAAGACAAGATTTGACCTAACCTCTAGAAGAGTTTGTCTAGAAGA

GTTATCCAATCAAATCTAAAAAATTCTACTTCAAAGTAGCAATAGCTCCAGGGTGTGACCACAAGGTTA

TGAAGCAGTCAGCACCATTGTCCCCGTGAA

>CLA_S_28_POT4625

TGTCCAATTTTGGAACCCTGCCTACAGCTGCTTCACTTTTAGGGAAGTTGATTTGGTACCTACCGTAGA

AGAGTATGTGGCTTTTCTACACTGCTCAAGGGTTAAAGTTGACAAGATCTATTCCAGAGTTGCCAACG

TCCCGCCCTTTGTAAAGAGACTGACGAGTATCACGGGGATGAGCGAGCAATGGATTGCGGCTAGGATT

AAGTAAAAGTGAGACAGAAGTTGTATTCCTTGGAAGAACCTAAGAGATCTAATCCTGGCACATCCGGA

TATGAAAAAGAGGGTTGATATTTTTGCT

>CLA_S_28_POT6578

CTTGTTTTTTAGTGTTGATCATTTGACGCTTTCCCCTGCATCTATCTTCTCACTTCTGATTGTATTTTCAA

TCATCTCGCCAAACATTACTATATCTGAGAAACTCTTCGTAGCGCTTCCCAACATCTGGTTAATGAATAG

GGCTTTCAGAGTGTTGATGAAAAGTATCATGGTTTCTTTCTCCAGAAGAGGTGGCTCGACTTGTGTCA

CTACCTCTCTCCATCTTTGGGCATATTGCTTGAAGCTCTCATTTTGCTTCTTTTTCATATTCTGCAGTGTA

ATTCTATCAGGTGTCATGTTT

>CLA_S_28_POT7818

ACATAACAATTTTTGGGGACGAAACTGGGGACAGAACTGCTTTCTTAACAATTTTAGTAGTTTTTTTTC

TATAATCTTTGCAAGAGCTGTCAAGGATGACAAGGTTACGAAATCTCCGTGCAGGAGGCAGAGTTGTT

GACAAATATCACAGAACATATGTTTGTTGACAAGGTTACGAAATCTCCATGCTGACAGTATTTTGTTAA

TTTTGCTGAAAGATCCAGATCTTGTTGAATTTCCTACATATAGACTTATGAAACGTTGTTGCTTTTGATA

TTTTAGGGTACACATGGTTTCATGA

>CLA_S_36_POT585

ATGCTGAGAGAACCGGCCAGCAATCTGATCTGAACAACTACTGCACAGGCCAAATAAAATTCCGAAA

GTGGAGCAATATTGTTAAAAGGAAAGTGATGTTAGAACCGGCAACTAGCCATAAACAGATGCTCCAAT

CATATCATTAAATAAACAATTGGAGAAAAAAAAAAGAAGAAGAGATAAAAAGATGCTTCAAGTCACTT

AGATAATCATGTCAGACGAGAATTTTATTTTTTCTTTTATTTCAGTTTTTTCATAGAGATGGAACTAAGC

AGATGGCATCTGATCAT

>CLA_S_36_POT4307

GGGTCTAAAACTGTTCTATGGTTGCAAACAAAGAGGACTCCACTTTGGCCTTTCCTAGGTGCTGGTGG

AGGGGTACCCTTAACAATCAATTTGATCCCCAGCAACCTGTAATTGTATCGAGCAATTCGCTCCGGCAA

CGGGATGTTGGTGTAGACCCTGAGTAGGGAGAGGATGAAGCCTATAGGAAGCCATAGGAAGGTCAAC

AGAGCAGCCAAAGGGGTTGGCCTTTGGACTAGGCGGCCCTCATGAAATATAACATGGCTTAGAAGCTT

GTTCCTTGGCAATGGTTCG

>CLA_S_36_POT8435

TGAAAACCTTACTTAGAATATATTTTAATTTTCAATTTTTTAATAACCAATCCCAGTCAAAGTGTGACTA

GACCATACTAGTTTCAAATAAAAAGGAGGTTTGCGTGCCAGAGGTAGTGGGAAGTTCGTGACGGCAA

CTGGGCAAAATTGAAGGGCATCGAGCACGGTTAATTAGAAGACAAAATAGGATAAATTAAGTAATGTG

TAAACATTTAGGACTATTTTTTTTTAAATTAATACTAAACTGACAAAACGTGTAAAGGTTAAGAGTTAA

ATTTGTTATTATGCTAA

>CLA_S_36_POT11074

AGCCAAATCACATGGCTTAGACTTATCACCACATGAACCAAAATCACCATCTCAAGTGAACATAACTAT

CATCTTTATAACTAGCCTATACATGCCATATTTACATATATCCACAAGTTCAGAAGTTCCAAACGGCAAC

TGGATAGTGTGATGCGTAACTCCGACTTGTCCAAAATGAGCGAGCTATCGAACCTCTATAAAACATGG

GAAAAGAAACAGAGTAAGCTTTCTAGCTTAGTAAGCCCGTATAGTTCAGAATTAAACTGACCATTTATA

CAATTAAGGCAA
